# Genetic Analysis of Potato Breeding Collection Using Single-Nucleotide Polymorphism (SNP) Markers

**DOI:** 10.3390/plants12091895

**Published:** 2023-05-06

**Authors:** Xi-ou Xiao, Ning Zhang, Hui Jin, Huaijun Si

**Affiliations:** 1 College of Agronomy, Gansu Agricultural University, Lanzhou 730070, China; xiao-forlearning@163.com (X.-o.X.); ningzh@gsau.edu.cn (N.Z.); 2 State Key Laboratory of Aridland Crop Science, Gansu Agricultural University, Lanzhou 730070, China; 3 South Subtropical Crop Research Institution, Chinese Academy of Tropical Agricultural Sciences, Zhanjiang 524091, China; jh3635315@sina.com; 4 College of Life Science and Technology, Gansu Agricultural University, Lanzhou 730070, China

**Keywords:** potato, genetic diversity, single-nucleotide polymorphism, SNP fingerprint

## Abstract

The autotetraploid potato (*Solanum tuberosum* L.) is an important crop in China, and it is widely cultivated from Northeast China to South China. Thousands of varieties are bred by breeding institutions or companies, and distinguishing the different varieties based on morphological characteristics is difficult. Using DNA fingerprints is an efficient method to identify varieties that plays an increasingly important role in germplasm identification and property rights protection. In this study, the genetic diversity and population structure of 135 autotetraploid potatoes were evaluated using specific-locus amplified fragment sequencing (SLAF-seq) methods. A total of 3,397,137 high-quality single-nucleotide polymorphisms (SNPs), which were distributed across 12 chromosomes, were obtained. Principal component analysis (PCA), neighbour-joining genetic trees, and model-based structure analysis showed that these autotetraploid potato subpopulations, classified by their SNPs, were not consistent with their geographical origins. On the basis of the obtained 3,397,137 SNPs, 160 perfect SNPs were selected, and 71 SNPs were successfully converted to penta-primer amplification refractory mutation (PARMS-SNP) markers. Additionally, 190 autotetraploid potato varieties were analysed using these 71 PARMS-SNP markers. The PCA results show that the accessions were not completely classified on the basis of their geographical origins. The SNP DNA fingerprints of the 190 autotetraploid potato varieties were also constructed. The SNP fingerprint results show that both synonyms and homonyms were present amongst the 190 autotetraploid potatoes. Above all, these novel SNP markers can lay a good foundation for the analysis of potato genetic diversity, DUS (distinctness, uniformity, and stability) testing, and plant variety protection.

## 1. Introduction

Potatoes (*Solanum tuberosum* L.) are the world’s fourth most important crop after rice, wheat, and maize. According to FAO data, the production of potatoes was 359 million tons across the world in 2021 (https://www.fao.org/faostat/en/#data/QI). The potato has an important role in food security, poverty alleviation, and improved health status because potatoes yield more food per unit of cropland in less time than any other major crop and are not at risk for the ill effects of speculative activity, unlike major cereal commodities [1]. Aside from providing basic nutrients, such as carbohydrates and dietary fibre, potatoes contain high nutritional value, such as vitamins, β-carotene, polyphenol, and minerals, that can improve human health [2,3].

Traditionally, potato genetic diversity is mainly estimated using morphological characteristics, such as plant height, number of stems per plant, characteristics of the stem, corolla colour, corolla shape, and total tuber yield ha^−1^ [4,5,6,7,8,9]. However, these characteristics are easily affected by the environment, field management, and some subjective factors. More importantly, most potatoes are similar in phenotype, making it difficult to distinguish them accurately. Thus, high-density genetic markers are meaningful and valuable for identifying and classifying different germplasms. DNA markers that are based on DNA structural or sequence variation are an effective tool to explore genetic variations in crop species. They can be easily detected in any tissue and growth stage in plants and they are not affected by the environment or pleiotropic and epistatic effects [10]. Different types of DNA markers, such as amplified fragment length polymorphisms (AFLPs) [11,12], simple sequence repeats (SSRs) [5,12], and SNPs [13,14,15,16,17], have been developed for potato genetic analysis. Because they are highly informative, codominant, and reproducible, SSR markers were one of the most widely used markers for plant genotyping as well as quantitative trait loci (QTL) mapping, genetic diversity analysis, DNA fingerprint defining, and so on [18]; however, the SSR process is time- and labour-consuming.

SNPs have been widely used in plant breeding, such as in genetic diversity analysis, QTL mapping, and molecular assistant selection, due to their automation for high-throughput and cost-effective genotyping [19,20,21,22]. There are three main approaches to identify SNPs in a plant genome: GBS, SNP arrays, and PCR-based methods. GBS and SNP arrays are powerful tools to genotype breeding populations that can identify large numbers (thousands to millions) of SNPs run in parallel [21]. Analysis of the genome-wide SNP of potatoes is simple and feasible because diploid and autotetraploid potato genome sequences can be dissected [23,24,25,26]. Several important QTLs of potatoes were mapped using GBS methods [26,27]. In the potato, several SNP arrays, such as 8 K, 10 K and 20 K SNPs, were developed [17,28]. These SNP arrays have been used to analyse potato genetic diversity, QTL mapping, and breeding history [13,14,15,16,17,28,29,30]. However, the processes of GBS and SNP arrays are also time-consuming. Most importantly, GBS and SNP arrays need expensive genotyping platforms and bioinformatic pipelines to analyse and interpret datasets [21,22]. So, GBS and SNP arrays are not suitable for most labs to analyse SNPs. PCR allelic discrimination technologies, such as TaqMan [19], Kompetitive Allele-Specific PCR (KASP) [31], and PARMS [32], have broad applications in the detection of SNPs in genetics. The PCR-based SNP detection approach is suitable for most labs because it is not costly and does not require special equipment. In recent years, hundreds of core SNP markers were discovered and used to analyse crop genetic diversity, as well as perform genome-wide association studies. In eggplants, 219 SNP markers were developed, and the unique DNA fingerprints of 377 eggplant varieties were established [33]. In pumpkins, 224 SNP markers were developed, and the genetic diversity of 223 cultivated pumpkin accessions was analysed [34]. In the potato, 25 KASP-SNPs markers were development and used for constructing a DNA fingerprint [35]. However, detailed information about the SNP markers was not published.

In this study, 135 potatoes were re-sequenced using SLAF-seq methods, and the resultant SNPs were called. Of these, 71 SNPs were successfully converted to PARMS-SNP markers based on their SLAF-seq results. These SNP markers could lay a good foundation for the analysis of potato genetic diversity, DUS testing, and plant variety protection.

## 2. Results

### 2.1. A Total of 3,397,137 SNPs Obtained by Genotyping-by-Sequencing (GBS)

**Figure 1:**
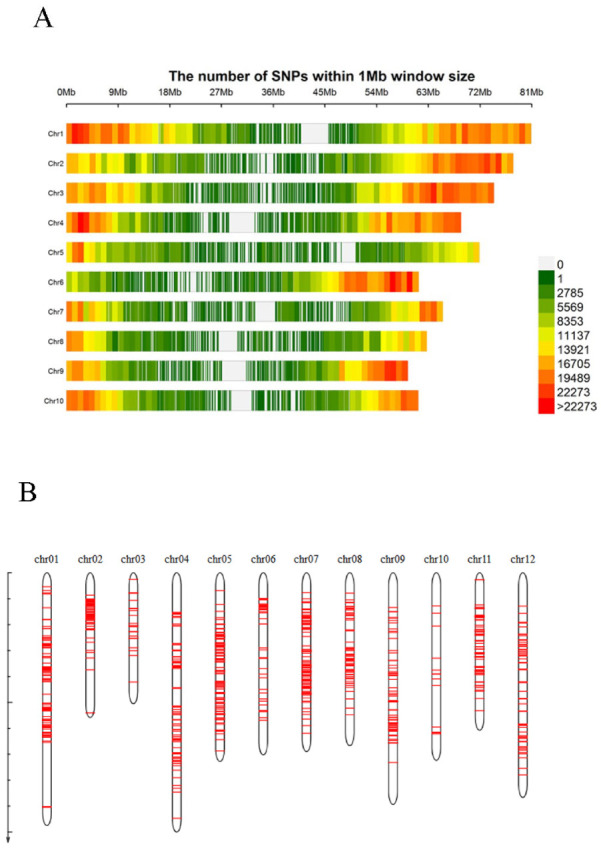
Distribution of 3,397,137 SNPs across 12 chromosomes, detected in 135 potatoes based on SLAF-seq. (**A**) Distribution of the 3,397,137 SNPs across 12 chromosomes. The bin size was 1 Mb. (**B**) The distribution of SNP-rich regions on the chromosomes. Counting the number of SNPs on the chromosome at a distance of 100 kb, the top 1% was labelled as SNP-rich. The physical positions of SNPs are based on the RHgv3 Haplotype I [26].

A total of 426.17 Mb of reads was obtained using SLAF-seq. After the reads were mapped to the referenced genome RHgv3 Haplotype I [26], 8,559,444 SNPs were called using SAMtools. Amongst them, the number of bi-allelic SNPs was 8,371,640 and the number of multiallelic SNPs was 187,804. This result indicates that although the 135 potatoes were autotetraploid, most of the SNPs were bi-allelic SNPs. After filtering was performed using the criteria of minor allele coverage read number > 3 and integrity > 85%, a total of 3,397,137 high-quality SNPs were obtained. These SNPs were well-distributed across 12 chromosomes, with the largest number of SNPs (396,991) found on chr01 and the fewest on chr03 (185,094, Figure 1A). In addition, the number of SNPs per 100 Kb along every chromosome was counted and the top 1% of regions in terms of the number of SNPs present was identified as SNP-rich [36]. Chr01 had the most SNP-rich regions, whereas chr10 had the fewest (Figure 1B). The 3,397,137 high-quality SNPs were used for phylogenetic tree construction, principal component analysis (PCA), and population structure analysis.

### 2.2. Structure and Genetic Diversity Analysis of 135 Potatoes

**Figure 2:**
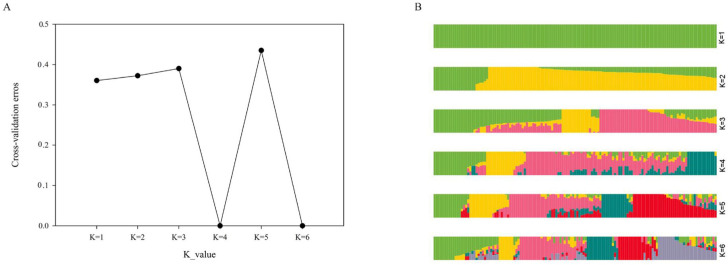
The model-based structure of 135 autotetraploid potatoes based on 3,397,137 SNPs. (**A**) The cross-validation errors; (**B**) the model-based structure of the 135 potatoes. Differently coloured segments indicate different populations. Green colour is presented by subgroup one, blue colour is presented by subgroup two, pink colour is presented by subgroup three, and yellow colour is presented by subgroup four.

The variation curve of the Bayesian information criterion value showed the optimal *K* value. The cross-validation error result indicates that the best *K*-value was *K* = 4 (Figure 2A). Based on their *Q* values, the 135 potatoes were classed into four subgroups. In subgroup one, a total of 25 potatoes were included, of which the geographical origin of 8 potatoes was Southwest China, the geographical origin of 3 potatoes was Northeast China, the geographical origin of 2 potatoes was Northwest China, the geographical origin of 2 potatoes was foreign, the geographical origin of 1 potato was North China, and the geographical origins of 8 potatoes were unknown. In subgroup two, a total of 19 potatoes were included, of which the geographical origin of 7 potatoes was Southwest China, the geographical origin of 4 potatoes was Central China, the geographical origin of 1 potato was Northwest China, the geographical origin of 1 potato was foreign, the geographical origin of 1 potato was North China, and the geographical origins of 5 potatoes were unknown. In subgroup three, a total of 77 potatoes were included, of which the geographical origin of 29 potatoes was Southwest China, the geographical origin of 3 potatoes was Northeast China, the geographical origin of 11 potatoes was Northwest China, the geographical origin of 4 potatoes was foreign, the geographical origin of 7 potatoes was North China, the geographical origin of 2 potatoes was Central China, and the geographical origins of 21 potatoes were unknown. In subgroup four, a total of 14 samples were included, of which the geographical origin of 6 potatoes was Southwest China, the geographical origin of 2 potatoes was Northeast China, the geographical origin of 2 potatoes was Northwest China, the geographical origin of 1 potato was foreign, and the geographical origins of 3 potatoes were unknown. The interlaced distribution of potato varieties shows that the differences in breeding areas are not necessarily related to genetic relationships.

**Figure 3:**
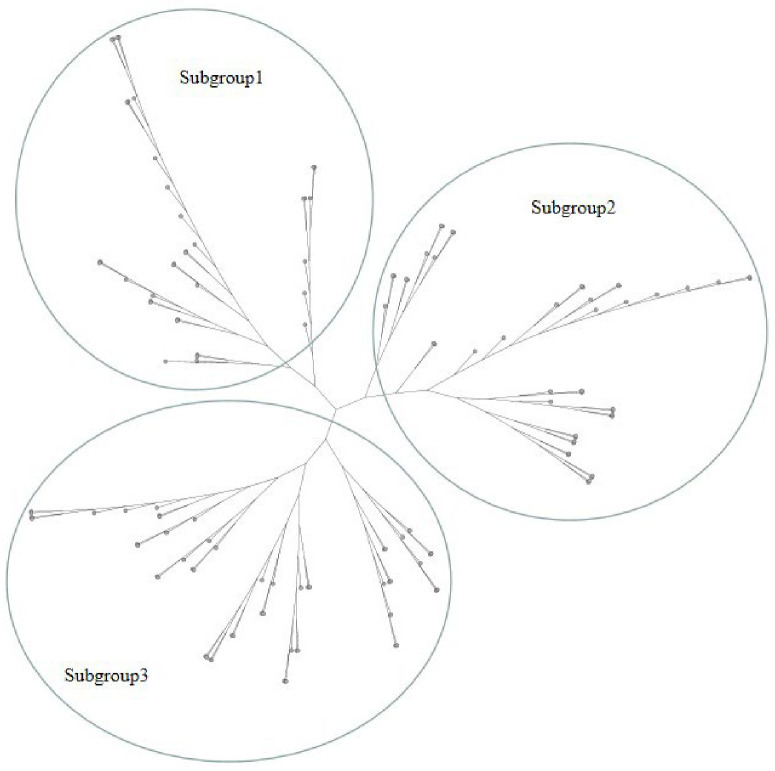
Neighbour joining (NJ) tree of 135 autotetraploid potato cultivars based on 339,713,771 SNPs.

**Figure 4:**
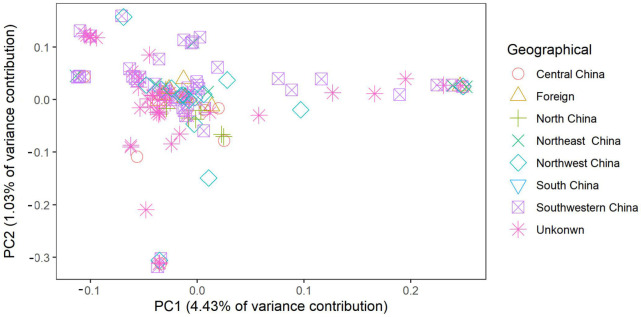
PCA of the 135 autotetraploid potato cultivars based on 3,397,137 SNPs. Different shapes and colours represent the potatoes’ geographical origins.

To analyse the relationship between the 135 potato varieties, a neighbour joining (NJ) cluster tree of 135 potatoes was constructed based on 339,713,771 SNPs (Figure 3). The result shows that the 135 potatoes were clearly classed into 3 subgroups. Subgroup one includes 36 potato varieties. Among the 36 potato varieties, the geographical origin of 18 potatoes was Southwest China, the geographical origin of 5 potatoes was Northeast China, the geographical origin of 5 potatoes was Northwest China, the geographical origin of 2 potatoes was Central China, and the geographical origins of 6 potatoes were unknown. Subgroup two includes 46 potatoes, of which the geographical origin of 8 potatoes was North China, the geographical origin of 8 potatoes was Southwestern China, the geographical origin of 6 potatoes was foreign, the geographical origin of 5 potatoes was Northwest China, the geographical origin of 3 potatoes was Central China, the geographical origin of 2 potatoes was Northeast China, and the geographical origins of 14 potatoes were unknown. Subgroup three includes 53 potatoes, of which the geographical origin of 24 potatoes was Southwestern China, the geographical origin of 6 potatoes was Northwest China, the geographical origin of 2 potatoes was Central China, the geographical origin of 2 potatoes was foreign, the geographical origin of 1 potato was North China, the geographical origin of 1 potato was Northeast China, and the geographical origins of 17 potatoes were unknown. PCA was conducted to assess the population structure. The first and second principal components explained 6.03% of the genetic diversity in total (Figure 4). The results show that the genetic diversity of these 135 potatoes is limited. Above all, these results indicate no correlation between geographical difference and genetic relationship.

### 2.3. Genome-Wide Perfect SNP Discovery in 135 Potato Cultivars

**Figure 5:**
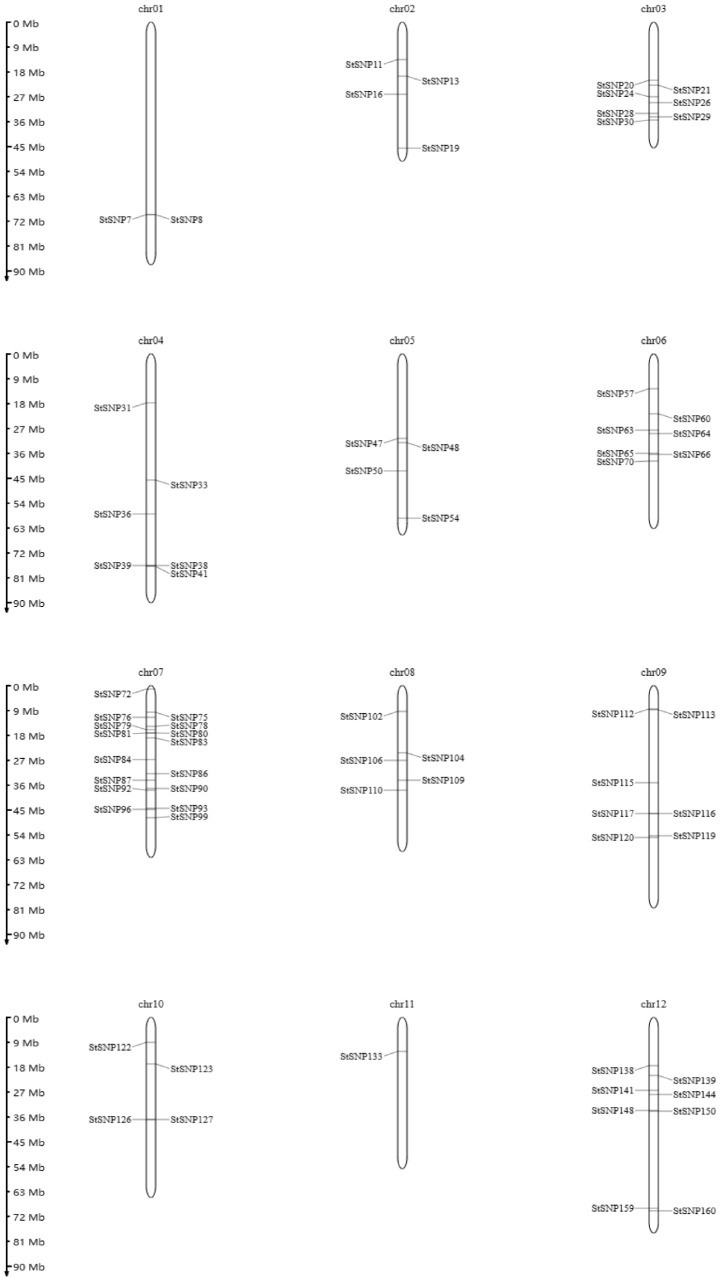
The distribution of 71 PARMS-SNP markers on the chromosomes. The physical positions of SNPs are based on RHgv3 Haplotype I [26].

After the 3,397,137 SNPs were filtered using (1) minor allele frequency (MAF) > 0.4, (2) miss rate < 0.2, (3) heterozygosity < 0.4, and (4) no sequence variation in the 100 bp flanking region, they were selected as perfect SNP candidates [33]. A total of 160 perfect SNPs were selected. However, only 71 SNPs were successfully converted to PARMS-SNP markers (Figure 5). Chr11 had only one PARMS-SNP marker, whereas chr07 contained the most, 16, PARMS-SNP markers.

### 2.4. Analysis of 190 Potatoes’ Genetic Diversity Based on 69 SNPs

After PCR amplification was conducted, the fluorescence values of FAM (5-carboxyfluorescein) and HEX (hexachlorofluorescein succinimide ester) were detected. The genotype was analysed using the fitploy R package on the basis of dosage score. Out of the 71 PARMS-SNP markers, 2 (StSNP63 and StSNP139) had one dosage class, while 6 PARMS-SNP markers had zero, one, two, three, four, and five dosage classes. Most of the PARMS-SNP markers had three dosage classes (Table 1). The mean “A” frequency (MA) of the 71 SNPs ranged from 1% to 100%, with a mean of 67.20%. Fifty-seven SNPs exhibited MAs higher than 50%. The observed heterozygosity (OH) of the 71 SNPs ranged from 0% to 100%, with a mean of 60%. The polymorphic information content (PIC) value of these SNPs ranged from 0 to 0.70, with a mean of 0.43. Given that the StSNP63 and StSNP139 markers had only one genotype, they were removed, and the remaining 69 StSNP dosage scores were used for the analysis of the 190 potatoes’ genetic diversity.

**Figure 6:**
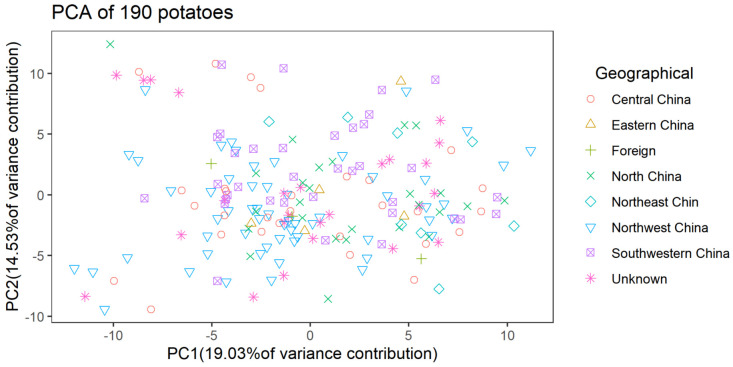
PCA of the 190 autotetraploid potato cultivars based on 69 PARMS-SNP markers. Different shapes represent samples from different geographic origins.

**Figure 7:**
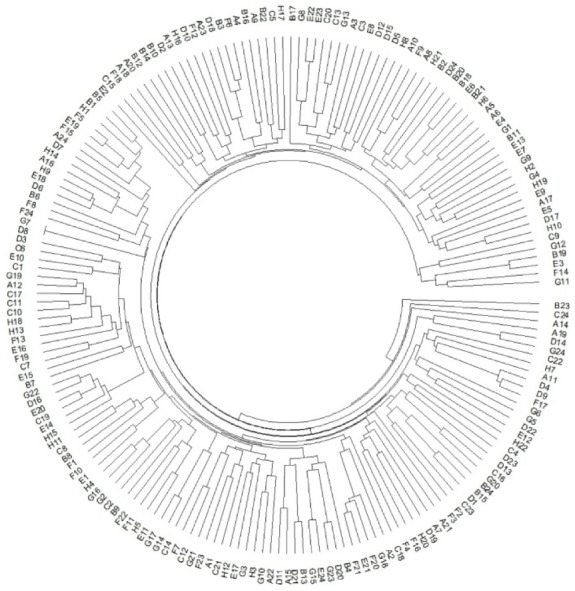
A UPGMA dendrogram constructed using the dosage scores of 69 SNP loci, showing all varieties differentiated, except for sample D8 and G7. Genetic distances were calculated with the ED distance.

The StSNP13 locus, with 48 samples, was detected using Sanger sequencing to assess the accuracy of the genotype result. The results show that the dosage score obtained in this manner may not give the correct genotype, as demonstrated (Table S1). However, dosage score is a robust and useful tool for the identification of tetraploid variety [37]. On the basis of these SNP dosage scores, the PCA of the 190 autotetraploid potatoes was performed using FactoMineR. The interlaced distribution of the autotetraploid potato varieties shows that the differences in breeding areas are not necessarily related to genetic relationships (Figure 6). On the basis of the dosage score, the genetic diversity of 190 autotetraploid potatoes was analysed. The results show that the genetic distance of pairs ranged from 0.00 to 9.00, indicating that that the genetic diversity was narrow. Meanwhile, a UPGMA dendrogram of the 190 potatoes was constructed (Figure 7). The results show that “Longshu No. 6” (sample ID: D8) and “Longshu No. 16” (sample ID: G7) were clustered together, whereas the other 188 potatoes were uniquely identifiable in the UPGMA dendrogram.

### 2.5. SNP Fingerprint Construction

**Figure 8:**
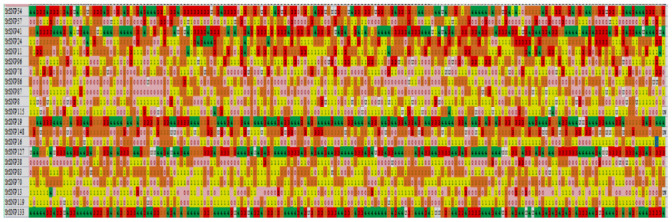
The SNP DNA fingerprint of the 190 potatoes based on the 21 PARMS-SNP markers. Each line means one sample, and each column means one SNP locus. Pink, yellow, orange, red, and green represent SNP dosage scores of 0, 1, 2, 3, and 4, respectively.

After SNP loci were selected in accordance with PIC values > 0.60, a total of 21 SNP loci were selected to construct the SNP fingerprints of the 190 autotetraploid potatoes. The results show that “Longshu No. 6” and “Longshu No. 16” were identical, whereas the other 188 potatoes were uniquely identifiable (Figure 8).

### 2.6. Identification of Variety Authenticity

**Figure 9:**
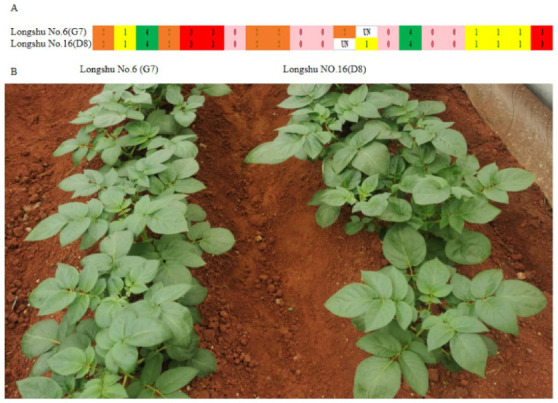
Identification of D8 and G7. (**A**) The SNP fingerprints of D8 and G7. Each line means one sample, and each column means one SNP locus. Pink, yellow, orange, red, and green represent SNP dosage scores of 0, 1, 2, 3, and 4, respectively. (**B**) Seedling phenotypes of D8 and G7 45 days after transplantation.

Different potato varieties could easily be mixed during transportation and conservation due to vegetative propagation. So, identifying variety authenticity is necessary. The UPGMA dendrogram shows that “Longshu No. 6” and “Longshu No. 16” were clustered together and that their SNP fingerprints were identical. The leaf morphological characteristics of the two samples were analysed to further verify the results and no differences were found between them, suggesting that the two samples were synonyms (Figure 9).

**Figure 10:**
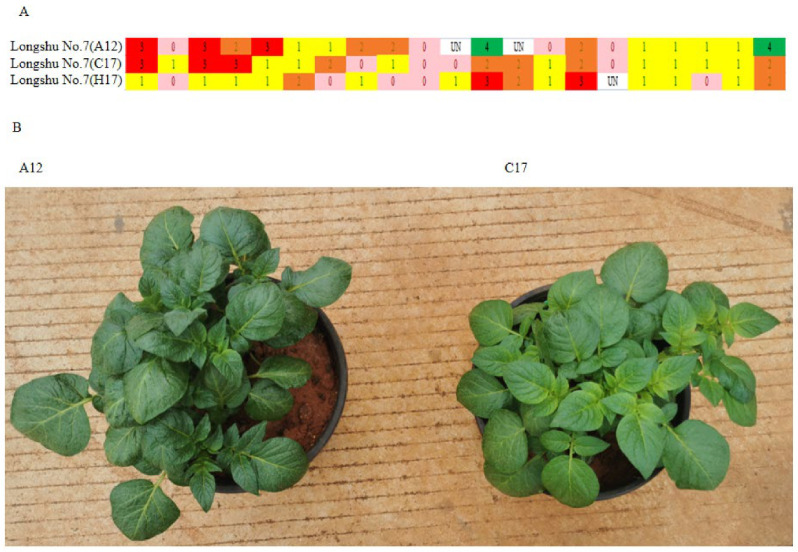
Identification of A12 and C17. (**A**) The SNP fingerprints of A12, C17, and H17. Each line means an SNP locus, and each column means samples. Pink, yellow, orange, red, and green represent SNP dosage scores of 0, 1, 2, 3, and 4, respectively. (**B**) Seedling phenotypes of A12 and C17 45 days after transplantation.

There were three samples named “Longshu No. 7” (sample IDs: A12, C17, and H17). However, the pairwise genetic distances between these samples were 6.61 (A12 and H17), 3.09 (A12 and C17), and 6.14 (C17 and H17). In addition, the UPGMA dendrogram shows that A12, C17, and H17 did not cluster together. The SNP fingerprints of the three potatoes were different (Figure 10A). The potato flesh colour of H17 was white, which was different from the potato flesh colours of A12 and C17, which were yellow. On this basis, the seedling phenotypes of A12 and C17 were compared. The results show that the leaf shapes and leaf colours of A12 and C17 were different (Figure 10B). These results suggest that A12, C17, and H17 were homonyms.

## 3. Discussion

Genotyping-by-sequencing (GBS) is a powerful approach to identify SNPs and analyse crop genetic diversity [38,39,40,41]. In the present study, the genetic diversity of 135 autotetraploid potato cultivars was analysed using SLAF-seq. Even though these varieties were autotetraploid, only 3% of the SNPs were multi-allelic SNPs, while the remaining 97% were bi-allelic. This result suggests that the genetic diversity of autotetraploid potatoes can be analysed on the basis of bi-allelic SNPs. In practice, several GBS methods and SNP arrays have been used to successfully analyse the genetic diversity of autotetraploid potatoes, in addition to being used for genome-wide association analysis [13,14,15,16,17,26,27,28,29,30,38]. The results could help us understand potato breeding history and molecular breeding. In the present study, the clustering results based on the SNPs show that the potatoes’ relationships were not consistent with geographical origin. This result is similar to the results of previous studies [42]. Some varieties shared the same parents, and it is also possible that some potatoes were introduced to one another by different research institutions in China. The potatoes with relationships were clustered together. In particular, “Holland No. 15” and Fiurita were clustered together via structure, PCA, and genetic trees because “Holland No. 15” was selected from a single plant of Fiurita.

GBS and SNP arrays are effective approaches to analyse genetic diversity, but they are costly. Several PCR-based methods, such KASP, TaqMan, and PARMS, are also effective in detecting SNPs. In the present study, 160 perfect SNPs were selected on the basis of 135 GBS data. However, only 43% of these perfect SNPs were successfully converted to SNP-PARMS markers. The low successful conversion ratio may be due to limited read depths (average sequencing depth is 10×) and the complex autotetraploid genome. Uitdewilligen et al. [41] showed that sequence depths of ∼60–80× could be used as a lower boundary for the reliable assessment of the allele copy numbers of sequence variants in autotetraploids.

PCR-based SNP identification methods, such as KASP, are widely used in diploids [31], but PCR-based SNP identification and utilisation for genotyping in polyploid species are limited due to their complex genomes and the lack of available SNP array genotype calls. Diploids only have three genotypes (AA, AB, and BB), and genotype calling for diploids can easily be achieved on the basis of FAM and VIC values [31]. However, autotetraploid potatoes have five genotypes, including nulliplex (AAAA), simplex (AAAB), duplex (AABB), triplex (ABBB), and quadruplex (BBBB). To the best of our knowledge, three programs can call the five genotypes on the basis of signal ratios: fitypoly (fitTetra) [43], ClusterCall [44], and superMASSA [45]. ClusterCall and superMASSA can only applied to SNP array data and not PCR-based signal ratios. Fitypoly (fitTetra) has been used for potato SNP genotyping on the basis of SNP dosage score [35,37]. In the present study, the genetic diversity of autotetraploid potatoes was analysed and the identification of their varieties was performed in accordance with SNP dosage scores. Our Sanger sequencing results show that the SNP dosage score result may not give the correct genotype, as demonstrated. The Sanger sequencing result could identify whether the SNPs were heterozygotes or homozygous. However, it could not identify whether the SNP heterozygotes were AAAB, AABB, or ABBB. Given that A/B are non-equally amplified during PCR and Sanger sequencing, the heterozygotes may have been incorrectly identified as homozygous; a similar result was obtained by Sasaki [37]. Despite being based on pyrophosphate sequencing, exactly determining the dosages of some SNP loci is difficult, but using SNP dosages is a simple, fast, and reliable tool for variety identification [37]. All four alleles in a potato are equally amplified by PCR and the nucleotide frequencies of the equally amplified alleles can accurately be correlated to their dosages [37]. Although the SNPs were selected as no-sequence variations in the 100 bp flanking region based on the 135 potato re-sequence data in the present study, the Sanger sequencing result shows two SNPs in the 5 bp flanking regions in some samples. Autotetraploid potatoes are highly heterozygotic and have abundant SNPs [37,41]. Some results indicate that the SNPs amongst As primers also affected the SNP genotyping results [34].

In this study, the genetic diversity of 190 autotetraploid potatoes genotyped using 69 SNP markers was analysed. The PCA results show that these potato clusters were not completely classified on the basis of their geographical origins. This result is consistent with the GBS result from the data of 135 potatoes. The pairwise genetic distance shows that the genetic diversity of the 190 potatoes was narrow because some potato varieties share the same parent or female relationships. So, extended genetic diversity is very important for autotetraploid potato breeding.

Autotetraploid potatoes are conserved or shared by tubers in the field or tissue culture seedlings in the lab, easily leading to confusion due to mislabelling and mechanical mixture [30]. DNA fingerprints were found to be an efficient method to identify true and false varieties. Sasaki [37] used 12 SNP loci to differentiate 115 potato varieties via pyrophosphate sequencing methods. In the present study, 21 SNP loci successfully differentiated 190 potatoes. This method is also more convenient and less costly. The pairwise genetic distance of “Longshu No. 6” and “Longshu No. 16” was 0 and their SNP fingerprints were identical. The leaf characters of the two samples were also identical. These results show that these two samples may be synonyms. However, the SNP fingerprints of three “Longshu No. 7” samples (sample IDs: A12, C17, and H17) differed, indicating that they were homonyms. The five samples were discarded, and other, correct, varieties were reintroduced.

Due to the complex breeding process, about 10 years is needed to develop new potato varieties. So, preventing unauthorised use of new varieties and support breeding activities is very important. Within the plant variety protection system, DUS testing is necessary. The current DUS testing process is labour-intensive, time-consuming, and environment-sensitive. Using DNA markers to supplement DUS testing is simple and efficient. These results could lay a solid foundation for potato DUS testing and plant variety protection.

## 4. Materials and Methods

### 4.1. Plant Materials and DNA Isolation

A total of 135 commercial autotetraploid potato cultivars (Table S2) were collected to identify genome-wide SNPs via SLAF-seq, and 190 samples (including autotetraploid cultivars and improving breeding line) were studied to establish genetic diversity fingerprints (Table S3). The total DNA was extracted from fresh true leaves following a cetyltrimethyl ammonium bromide (CTAB) method. The DNA concentration and quality of all samples were assessed with a Nanodrop 2000 UV (NanoDrop, Wilmington, DE, USA). The quantified DNA was diluted to 100 ng·μL^−1^ for SLAF-seq and 20 ng μL^−1^ for PARMS-SNP genotyping.

### 4.2. Genotyping by SLAF Sequencing

After the DNAs were digested using *Hae* III-*Hpy*166II, GBS was performed for 135 cultivars following the protocol of Sun et al. [46]. The libraries were sequenced with the pair-end method using the HiSeq 2500 platform. After the reads were filtered using fastq software under the default parameters, the clean reads were mapped to the heterozygous diploid potato genome RHgv3 Haplotype I [26] by using Burrows–Wheeler alignment with the default parameters. SNP calling was conducted using SAMtools. Ultimately, after the multiallelics were filtered, the consistent SNPs were selected using the following criteria: minor allele coverage read number > 3 and integrity > 85%. The SNP density was analysed using the CMplot R pakcage with a 1 Mb bin size.

### 4.3. Phylogenetic Tree Construction and PCA

On the basis of the 3,397,137 identified SNPs, the genetic distances amongst 135 potatoes were analysed using neighbour-joining and polygenetic trees, which were constructed using Phylip [47]. The polygenetic tree was modified using the online tool Itol (https://itol.embl.de). PCA was conducted using Plink2 (https://www.cog-genomics.org/plink/2.0/).

### 4.4. SNP Genotyping via PARMS

The detected SNPs were filtered using the following criteria: (1) MAF > 0.4, (2) miss rate < 0.2, (3) heterozygosity < 0.4, and (4) no sequence variation in the flanking region of 100 bp. SNPs that fulfilled these were selected as perfect SNP candidates [33]. The primers were designed using the online software snpway (www.snpway.com). The primers are listed in Table S4.

Genotyping tests were carried out with a 5 μL PCR reaction system and the thermal cycling program of PARMS in accordance with Lu et al. [32]. PCR amplification was performed and the fluorescence values of FAM and HEX were detected using an ABI QuantStudio 6 Flex real-time PCR instrument. The genotype dosage score was analysed using the fiypoly R package with the parameters of p.threshold = 0.6 and peak.threshold = 1.

### 4.5. Data Analysis

After the 190 potatoes were genotyped using 71 SNP markers, PCA was performed using the FactoMineR R package [48].

Each SNP locus and variety were characterised by MA, OH, and PIC. MA was calculated as the proportion of the amount of “A” compared with the sum of all genotype calls for each sample, not including SNPs with no calls. OH was calculated as the proportion of heterozygous genotypes (AAAB, AABB, and ABBB) compared with the sum of all genotype calls for each sample, not including SNPs with no calls. PIC was analysed following the description of Sasaki [37]. Dendrograms were constructed from the allele dosage scores of the SNP markers via hierarchical cluster analysis of the pairwise ED distances using the hclust package in the stats R package and the UPGMA method. The SNP fingerprints were constructed on the basis of SNPs with PIC > 0.6.

## 5. Conclusions

In this study, the genetic diversity of autotetraploid potatoes was analysed using GBS and SNP markers. The results demonstrate that SNP markers are a powerful tool to detect specific loci and/or alleles in autotetraploid potatoes. On the basis of the GBS data, 69 SNP markers were developed, and the genetic diversity of 190 autotetraploid potatoes was analysed using the 69 SNP markers. The SNP fingerprints of the 190 autotetraploid potatoes were also constructed. These novel 69 SNP markers could lay a solid foundation for the analysis of potato genetic diversity, DUS testing, and plant variety protection.

## Figures and Tables

**Table 1:** Evaluation of each SNP locus by the number of varieties in each of five SNP genotypes.

Marker Name	SNPs Dosage Score						OH	MA	PIC
	0	1	2	3	4	NA			
StSNP54	4	11	52	71	39	15	0.76	0.32	0.70
StSNP57	78	46	43	18	0	7	0.58	0.75	0.70
StSNP41	0	15	36	69	60	12	0.67	0.26	0.70
StSNP24	13	64	77	29	7	2	0.89	0.56	0.69
StSNP11	11	65	78	30	6	2	0.91	0.56	0.69
StSNP96	32	88	41	23	0	8	0.83	0.68	0.68
StSNP78	70	70	28	14	0	10	0.62	0.77	0.67
StSNP86	72	42	66	1	0	11	0.60	0.76	0.65
StSNP87	74	70	39	2	0	7	0.60	0.79	0.65
StSNP8	48	85	38	5	0	16	0.73	0.75	0.64
StSNP115	64	83	25	5	3	12	0.63	0.78	0.64
StSNP19	0	0	41	62	85	4	0.55	0.19	0.64
StSNP148	23	23	90	31	0	25	0.86	0.56	0.64
StSNP16	75	77	34	0	0	6	0.60	0.81	0.63
StSNP127	0	3	27	75	74	12	0.59	0.19	0.63
StSNP38	91	62	29	5	0	5	0.51	0.82	0.63
StSNP83	32	84	71	0	0	5	0.83	0.70	0.62
StSNP70	35	93	62	0	0	2	0.82	0.71	0.62
StSNP13	70	76	17	6	1	3	0.58	0.81	0.62
StSNP119	67	89	31	0	0	5	0.64	0.80	0.62
StSNP133	0	1	24	69	85	13	0.53	0.17	0.61
StSNP80	78	89	23	0	0	2	0.59	0.82	0.60
StSNP39	39	104	46	0	0	3	0.79	0.74	0.60
StSNP21	71	94	22	0	0	5	0.62	0.82	0.59
StSNP30	64	97	20	1	0	9	0.65	0.81	0.58
StSNP26	40	109	39	1	0	3	0.79	0.75	0.58
StSNP33	83	82	16	0	0	11	0.54	0.84	0.58
StSNP109	58	101	20	0	0	13	0.68	0.80	0.56
StSNP106	61	103	17	1	0	10	0.66	0.81	0.56
StSNP112	102	67	13	2	0	8	0.45	0.87	0.55
StSNP76	19	109	60	0	0	4	0.90	0.70	0.55
StSNP104	61	105	15	0	0	11	0.66	0.81	0.54
StSNP90	114	51	20	2	0	4	0.39	0.87	0.54
StSNP110	115	47	13	9	0	8	0.38	0.86	0.54
StSNP141	34	114	32	0	0	12	0.81	0.75	0.53
StSNP99	0	2	6	66	96	22	0.44	0.12	0.53
StSNP7	26	118	34	0	0	14	0.85	0.74	0.50
StSNP93	25	117	34	0	0	16	0.86	0.74	0.50
StSNP84	0	4	14	118	35	20	0.80	0.23	0.47
StSNP47	130	42	7	5	0	8	0.29	0.90	0.45
StSNP72	0	55	126	0	0	11	1	0.58	0.42
StSNP92	2	131	31	14	0	14	0.99	0.67	0.42
StSNP81	0	0	15	28	132	17	0.25	0.08	0.40
StSNP66	5	140	41	0	0	6	0.97	0.70	0.38
StSNP113	7	140	38	0	0	7	0.96	0.71	0.38
StSNP120	13	145	31	0	0	3	0.93	0.73	0.38
StSNP65	0	1	135	44	0	2	1	0.44	0.38
StSNP75	0	0	1	37	150	4	0.20	0.05	0.32
StSNP159	144	33	1	0	0	14	0.19	0.95	0.31
StSNP160	30	154	2	0	0	6	0.84	0.79	0.29
StSNP102	160	22	7	0	0	3	0.15	0.95	0.27
StSNP123	149	26	0	1	0	16	0.15	0.96	0.26
StSNP126	150	26	0	1	0	15	0.15	0.96	0.26
StSNP36	29	160	0	0	0	3	0.85	0.79	0.26
StSNP122	15	168	7	0	0	2	0.92	0.76	0.21
StSNP48	0	0	0	22	166	4	0.12	0.03	0.21
StSNP28	167	22	0	0	0	3	0.12	0.97	0.21
StSNP29	163	19	0	0	0	10	0.10	0.97	0.19
StSNP31	169	16	3	0	0	2	0.10	0.97	0.18
StSNP64	175	15	0	0	0	2	0.08	0.98	0.15
StSNP60	176	14	0	0	0	2	0.07	0.98	0.14
StSNP50	0	180	10	0	0	2	1	0.74	0.10
StSNP116	2	172	5	2	0	11	0.99	0.74	0.10
StSNP144	0	0	0	8	181	3	0.04	0.01	0.08
StSNP138	0	0	0	8	182	2	0.04	0.01	0.08
StSNP20	4	182	2	0	0	4	0.98	0.75	0.06
StSNP117	0	2	177	3	0	10	1	0.50	0.05
StSNP150	185	5	0	0	0	2	0.03	0.99	0.05
StSNP79	1	188	1	0	0	2	0.99	0.75	0.02
StSNP63	190	0	0	0	0	2	0	1	0
StSNP139	0	0	190	0	0	2	1	0.50	0

Note: 0, 1, 2, 3, and 4 represent different genotypes of AAAA, AAAB, AABB, ABBB, and BBBB.

## Data Availability

No data need to be shared.

## Supplementary Materials

The following supporting information can be downloaded at: https://www.mdpi.com/article/10.3390/plants12091895/s1. Table S1: The Sanger sequencing result of the StSNP138 locus. Table S2: The 135 potatoes used for SLAF-seq. Table S3: The 190 potatoes were analysed using 71 PARMS-SNP markers. Table S4: The primers of the 71 PARMS-SNP markers.
