# Supplementary material for: Genetic Analysis of Potato Breeding Collection Using Single-Nucleotide Polymorphism (SNP) Markers
Source: Plants (Basel). 2023 May 6;12(9):1895. doi: 10.3390/plants12091895 (PMC10181131; doi:10.3390/plants12091895)
Supplement: Supplementary file 1 [file plants-12-01895-s001.zip › Table S1.pdf]

Table S1. Sanger sequence result of the StSNP138.

Sample	Dosage score	Genotype	Sanger sequence result
A7	1	CCCT	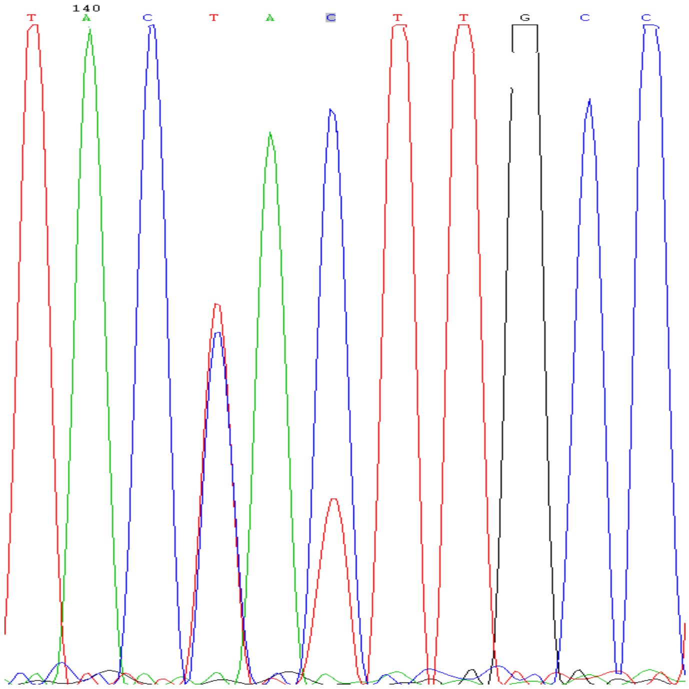
A22	2	CCTT	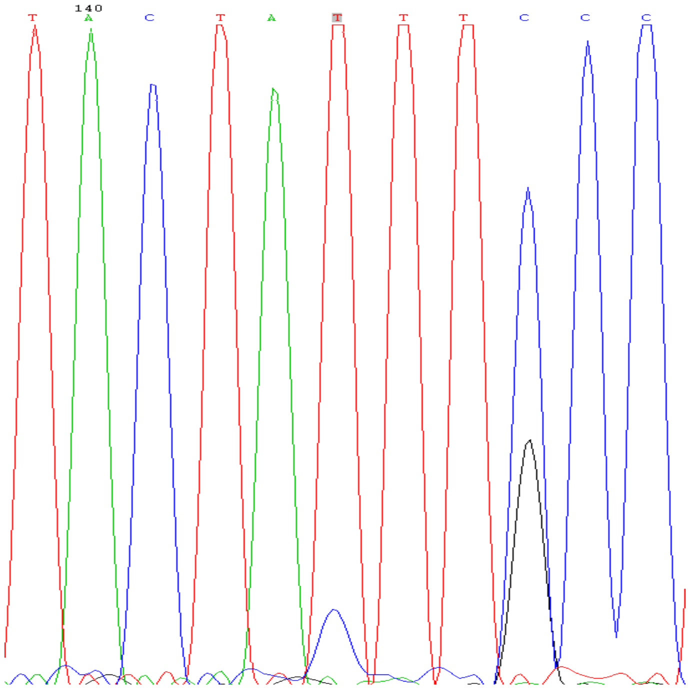

A23	0	CCCC	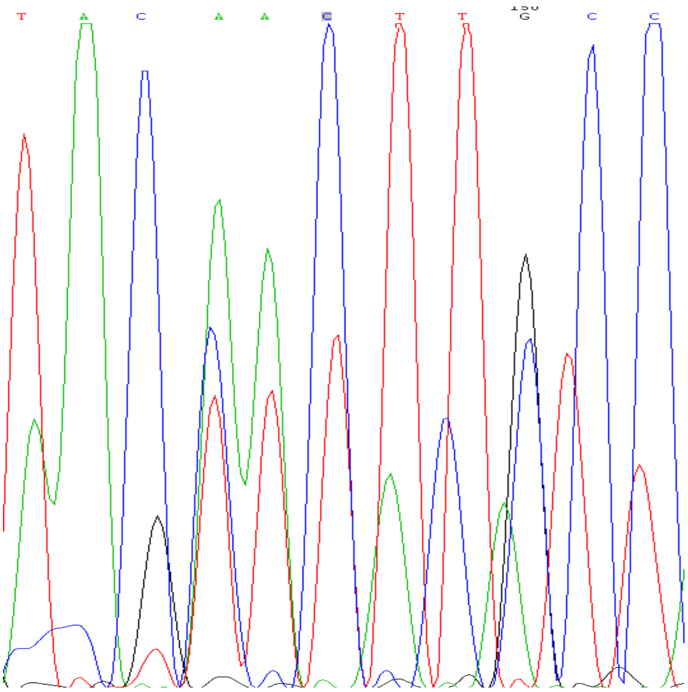
B7	UN	UN	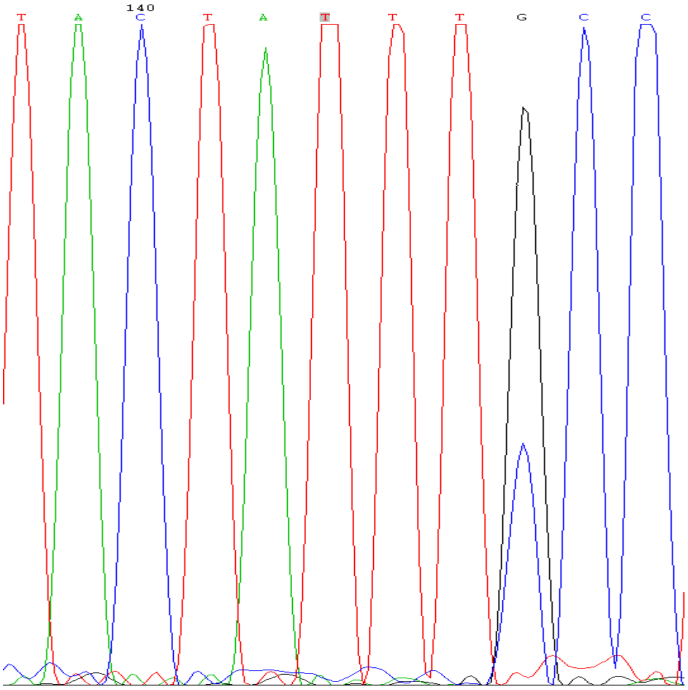

B9	0	CCCC	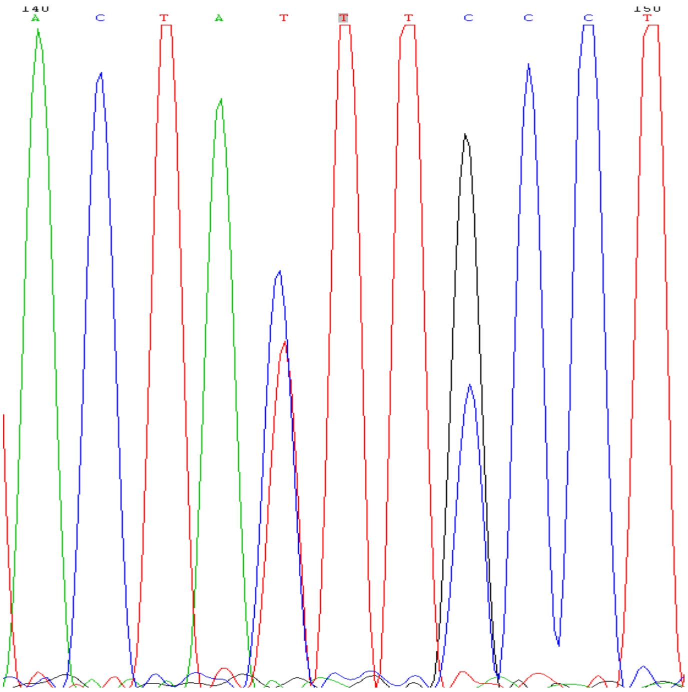
B12	2	CCTT	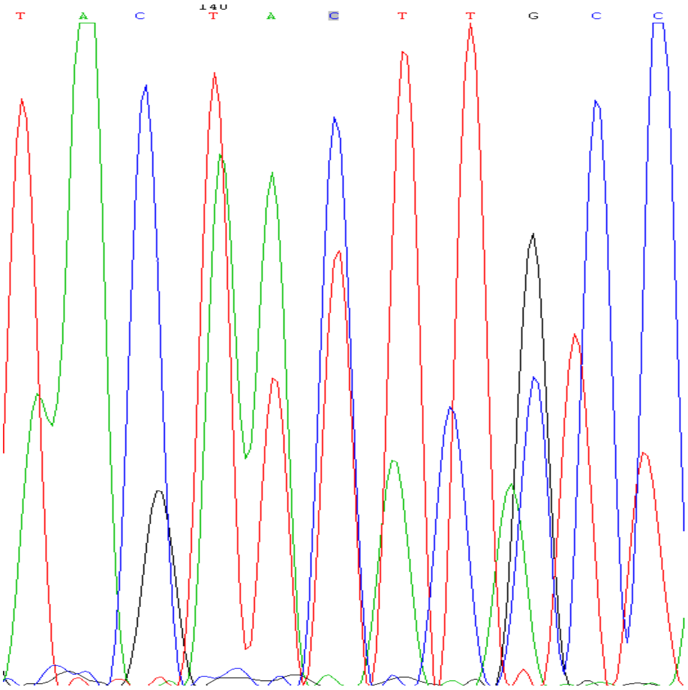

H7	0	CCCC	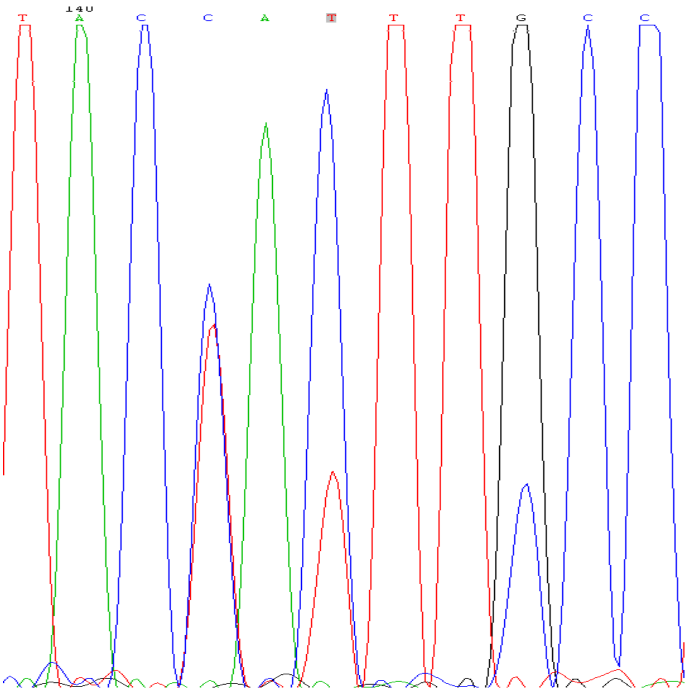
G5	UN	UN	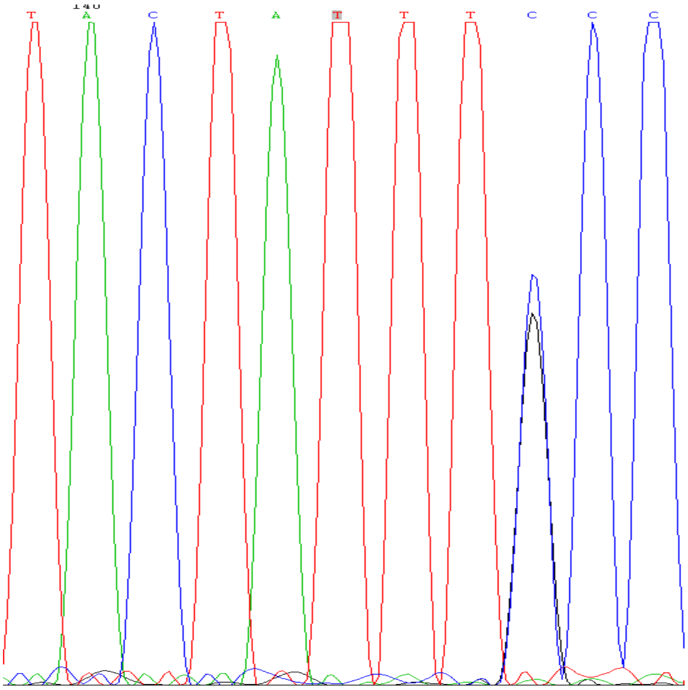

H3	2	CCTT	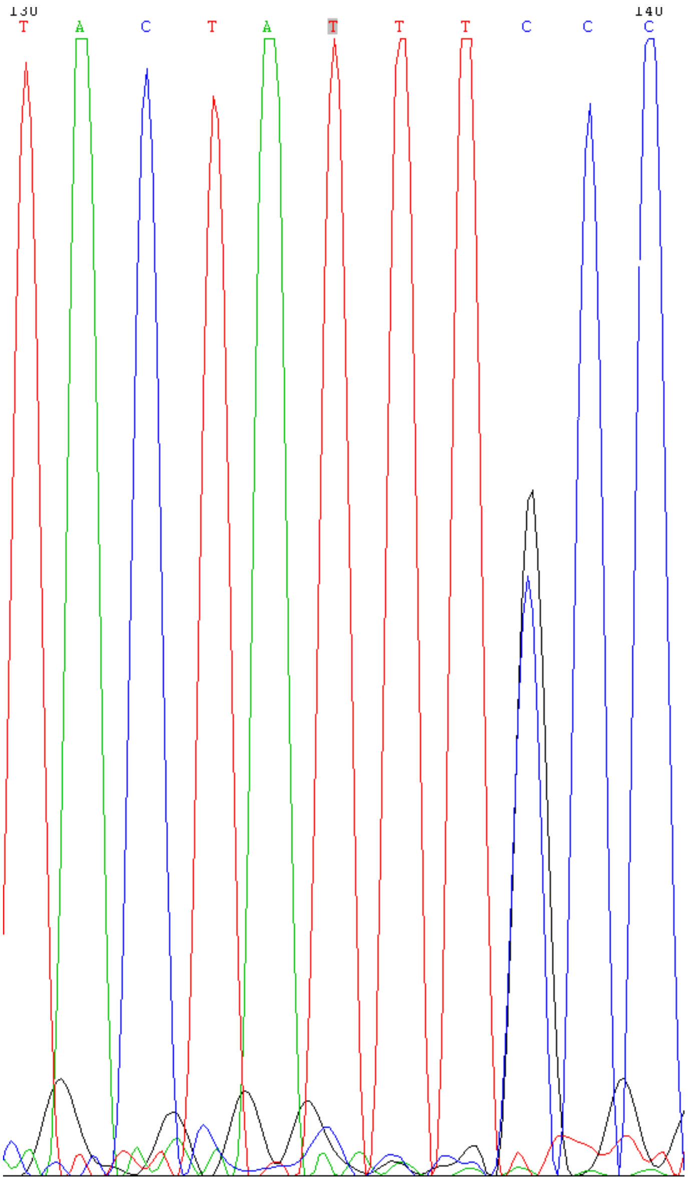

G10	3	CTTT	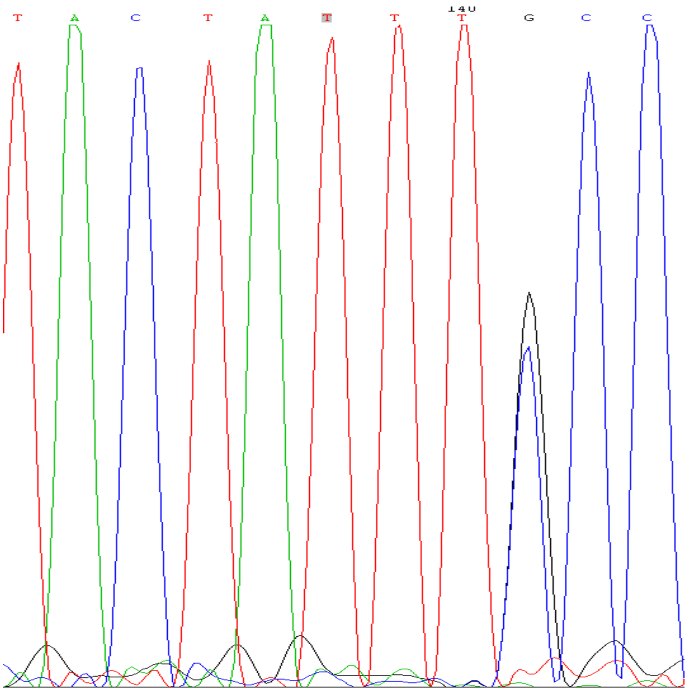
D11	3	CTTT	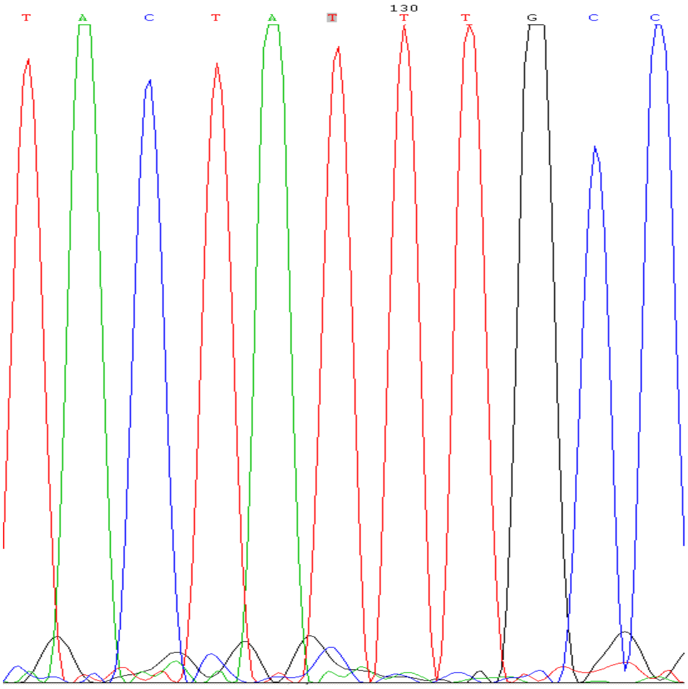

C13	UN	UN	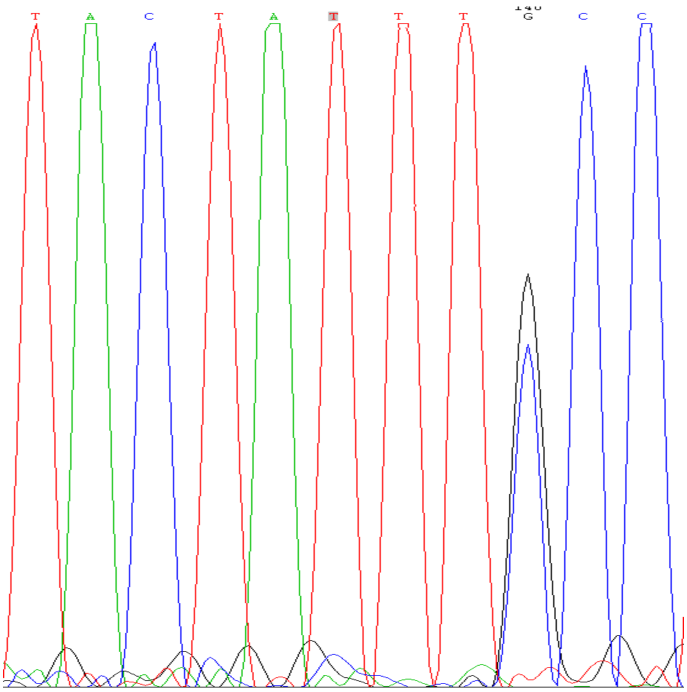
F13	UN	UN	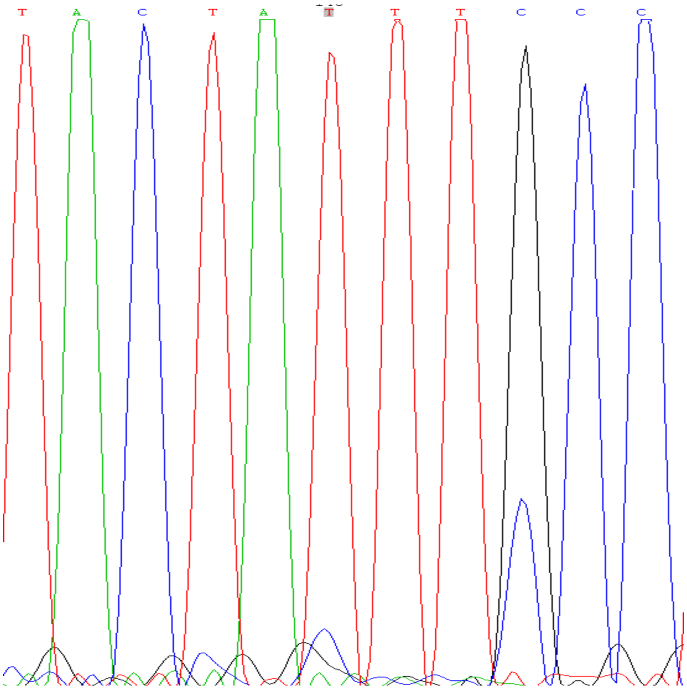

G13	3	CTTT	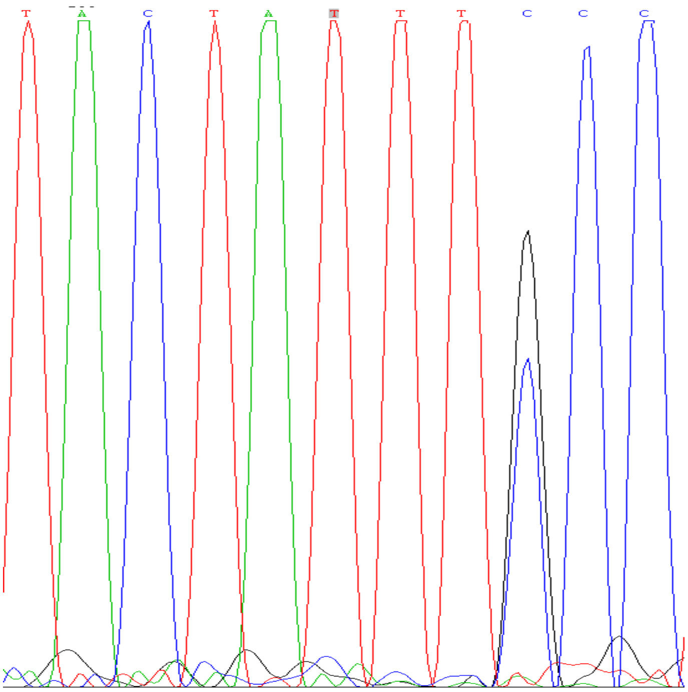
D1	0	CCCC	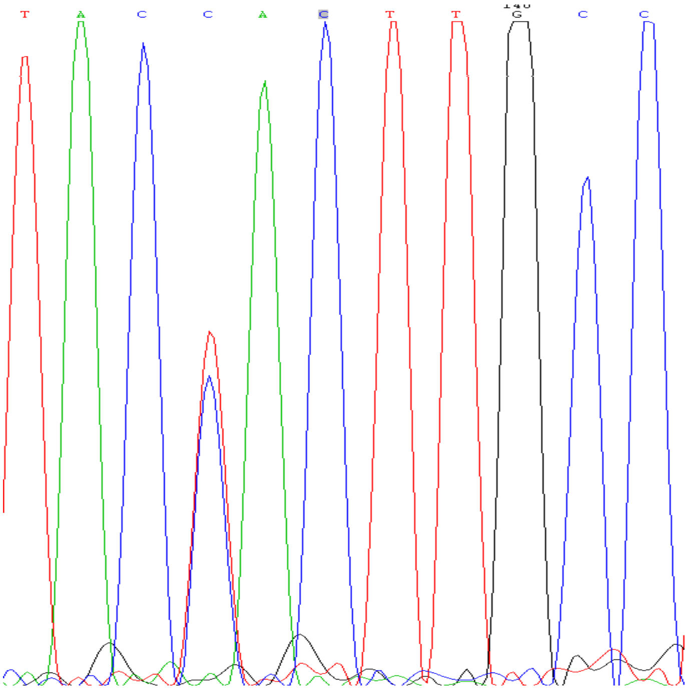

F1	0	CCCC	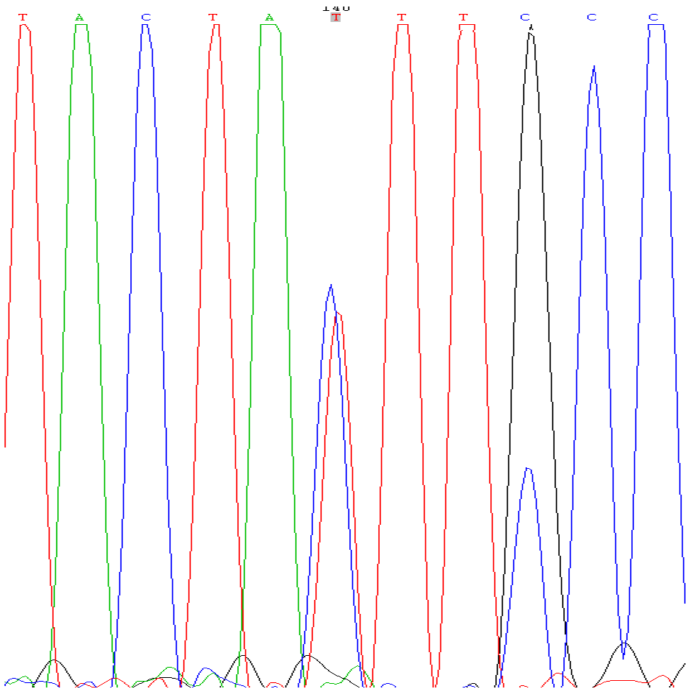
G1	0	CCCC	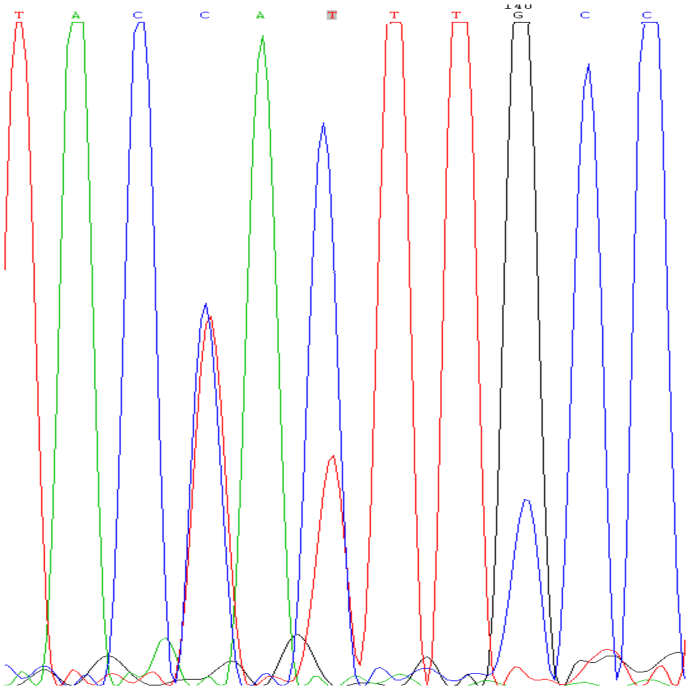

E1	0	CCCC	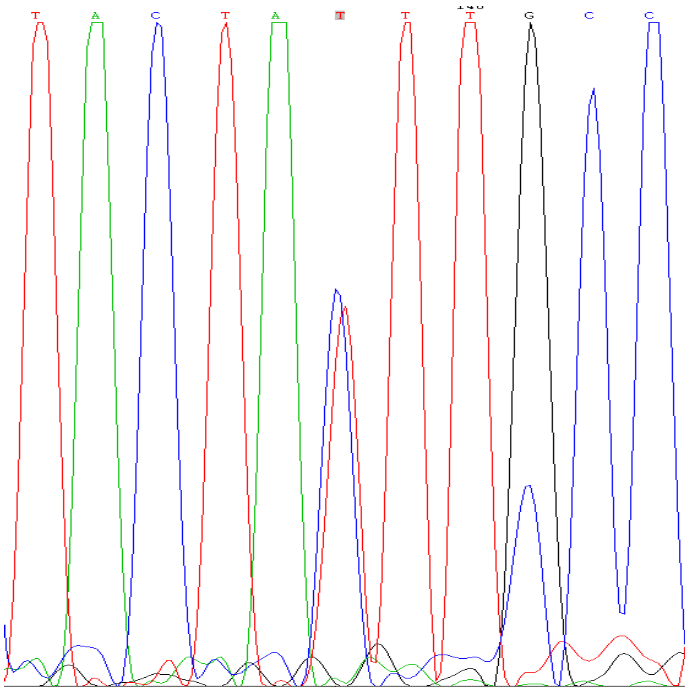
E2	0	CCCC	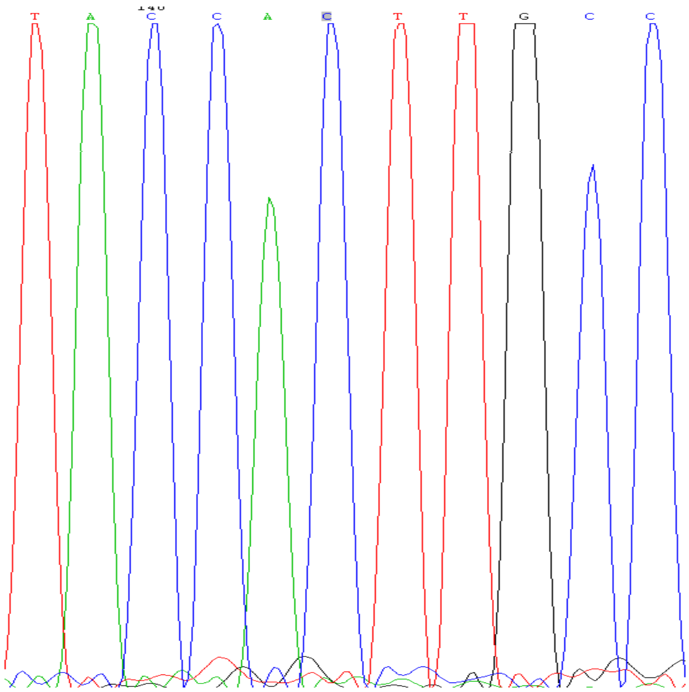

E3	1	CCCT	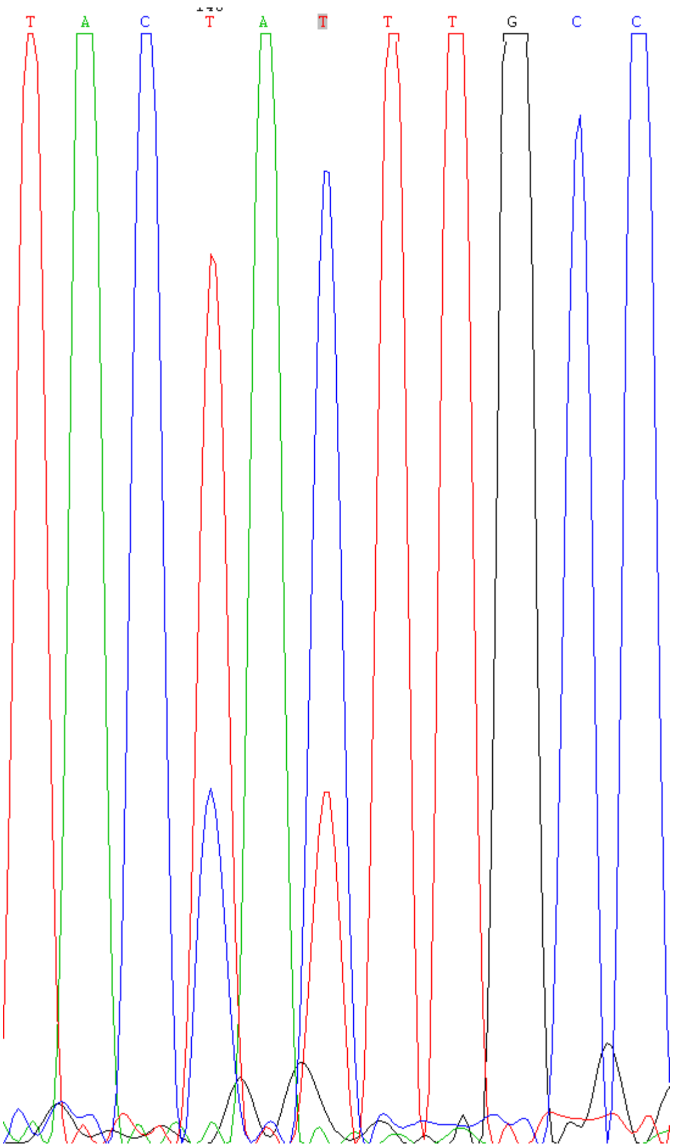

E4	1	CCCT	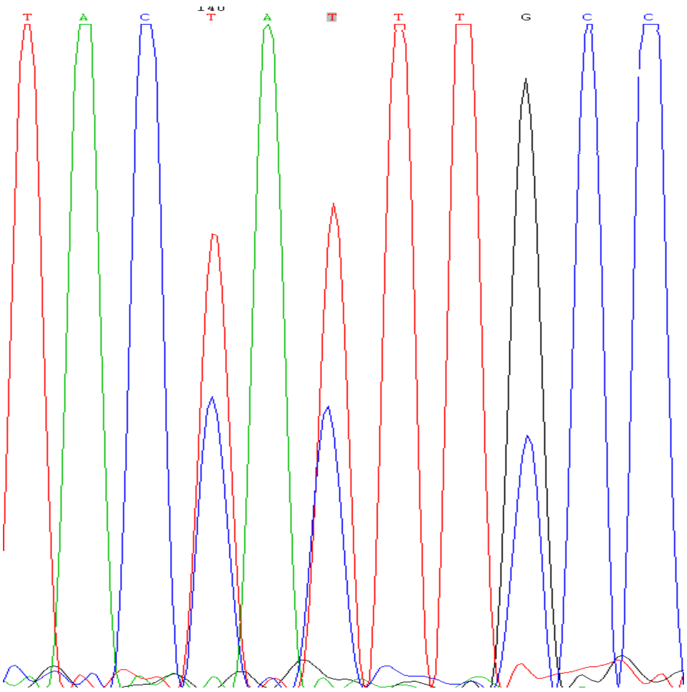
E5	2	CCTT	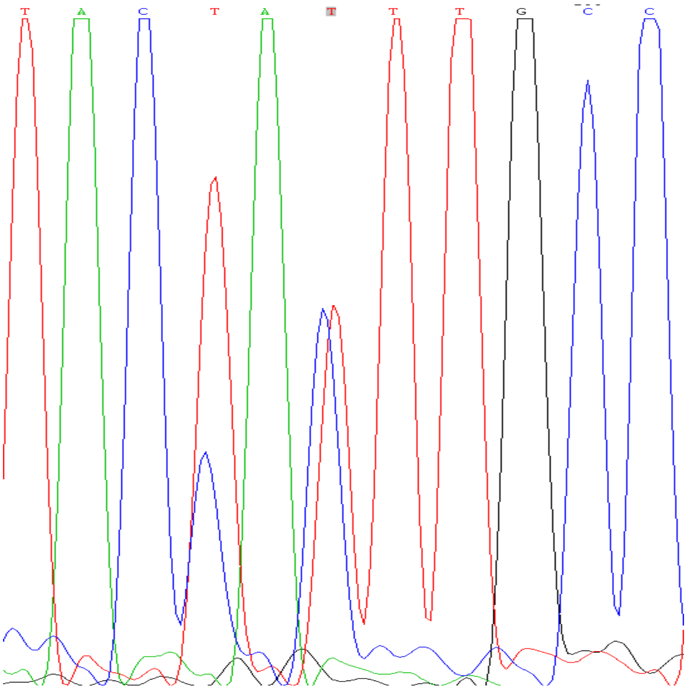

E6	0	CCCC	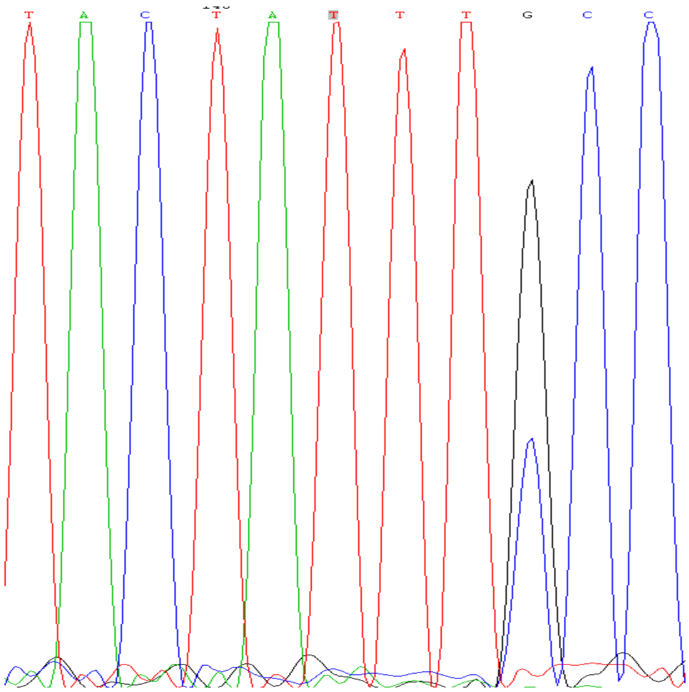
E7	0	CCCC	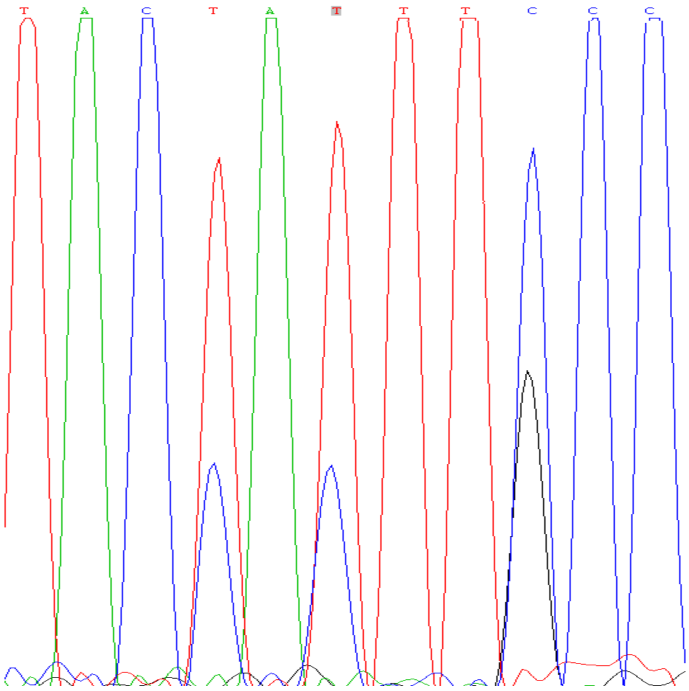

E8	1	CCCT	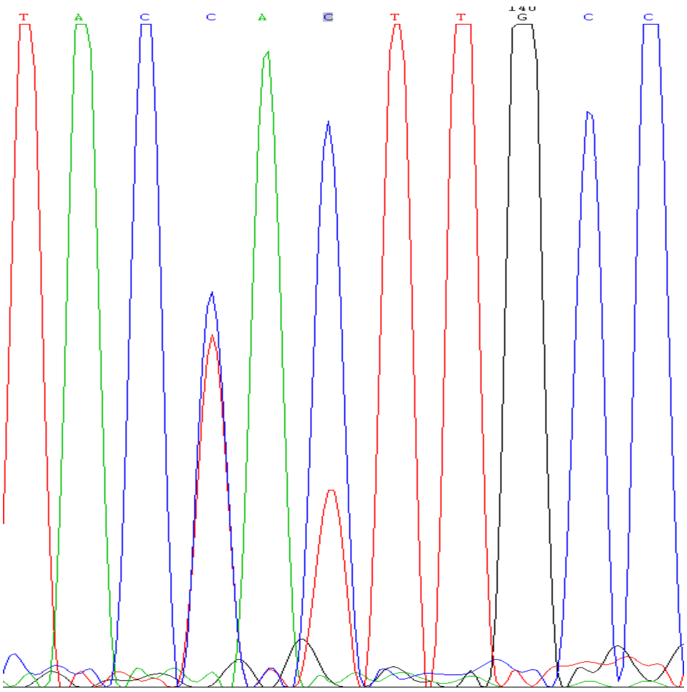
E9	0	CCCC	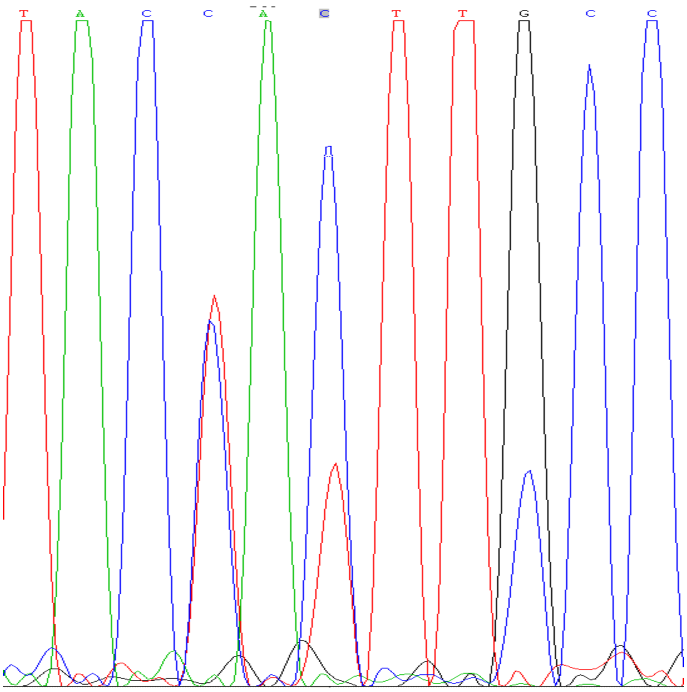

E10	2	CCTT	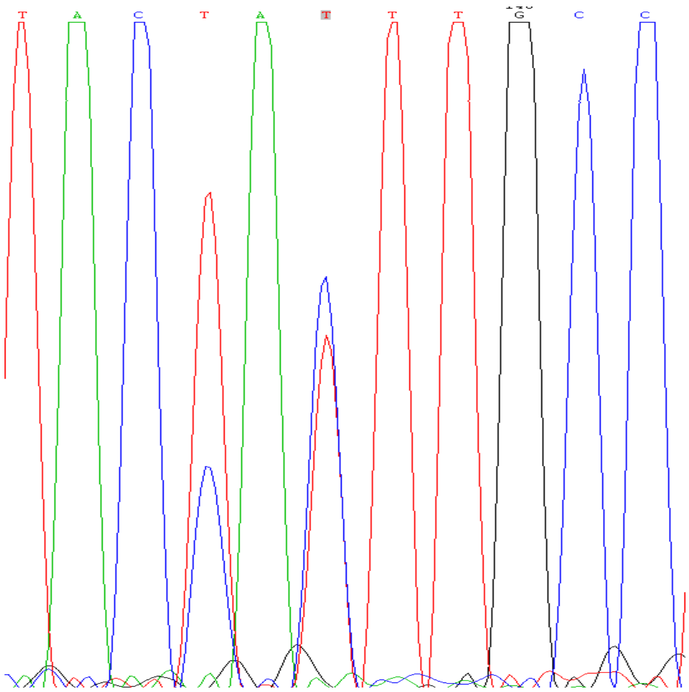
E11	0	CCCC	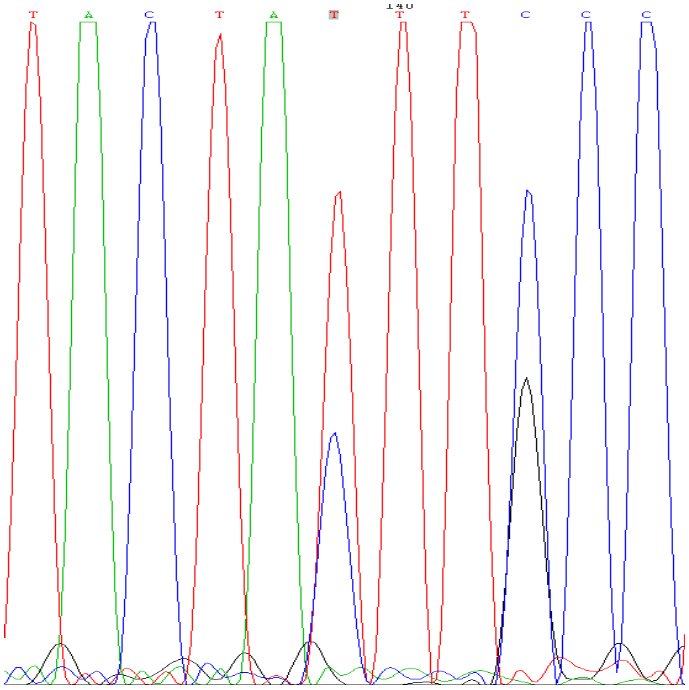

E12	0	CCCC	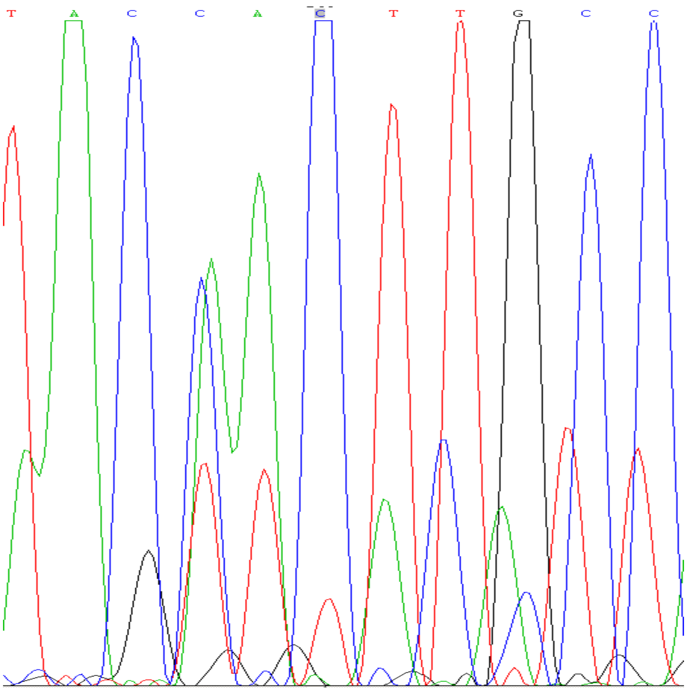
E13	0	CCCC	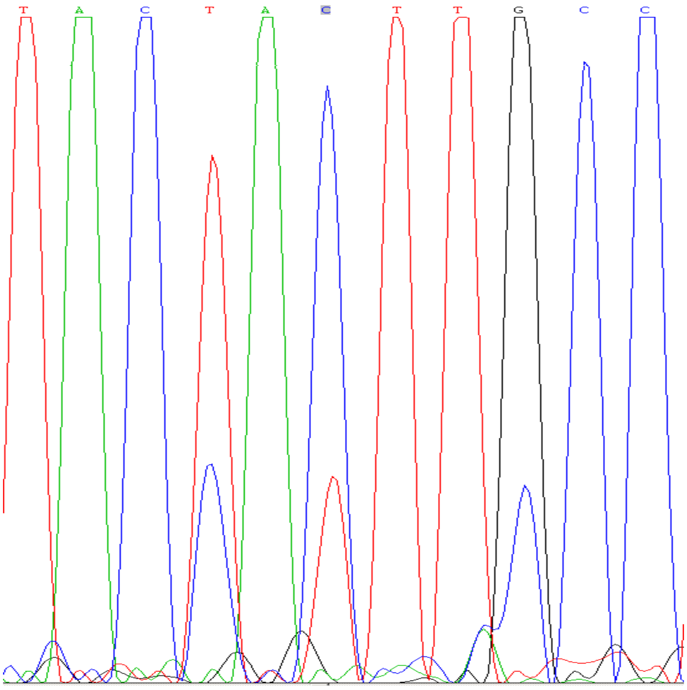

E14	2	CCTT	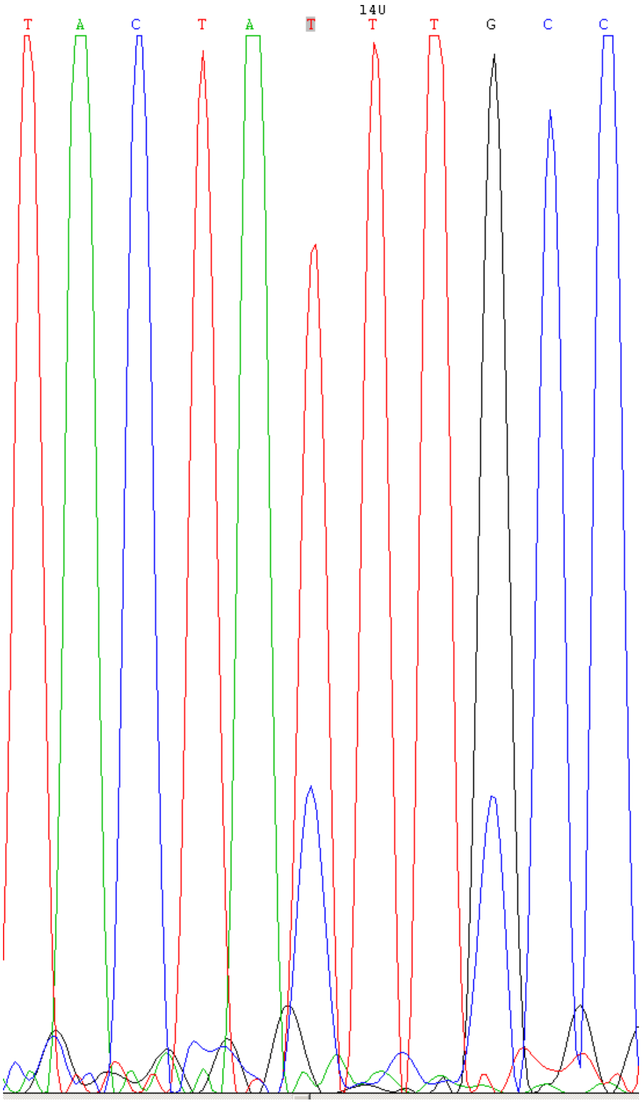
E15	3	CTTT	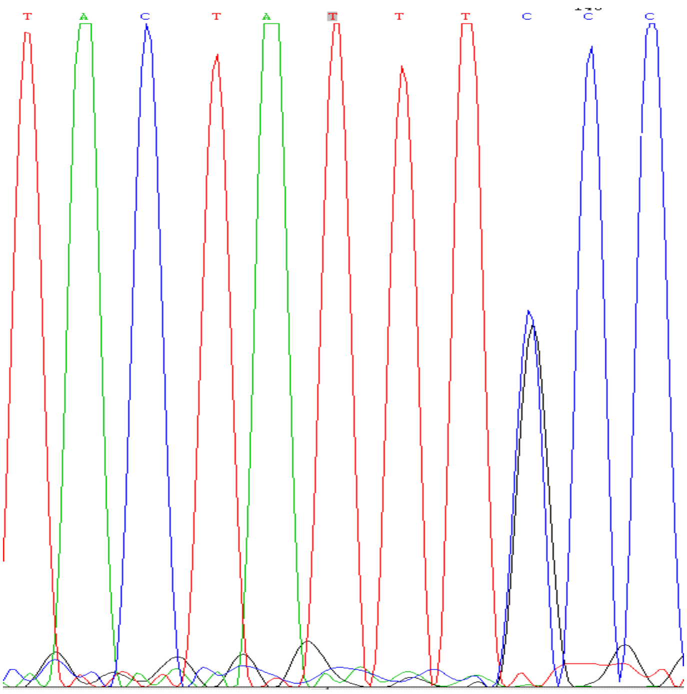

E16	3	CTTT	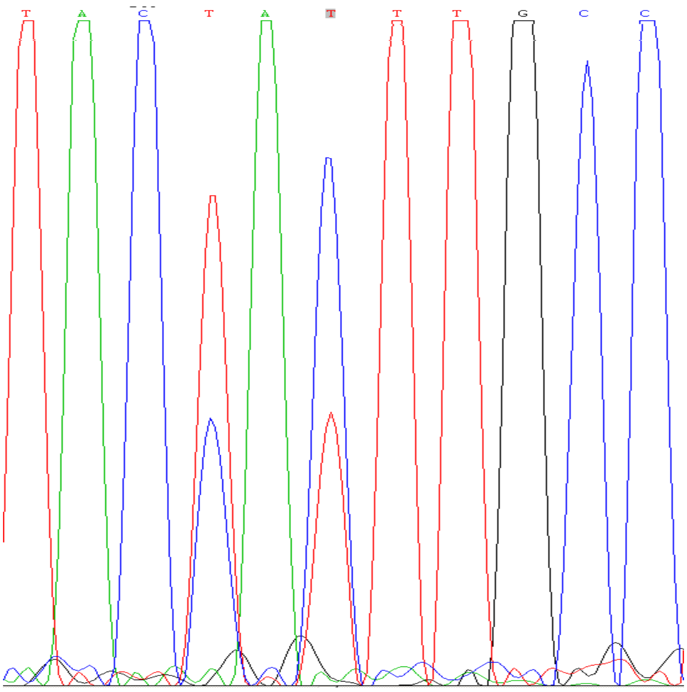
E17	0	CCCC	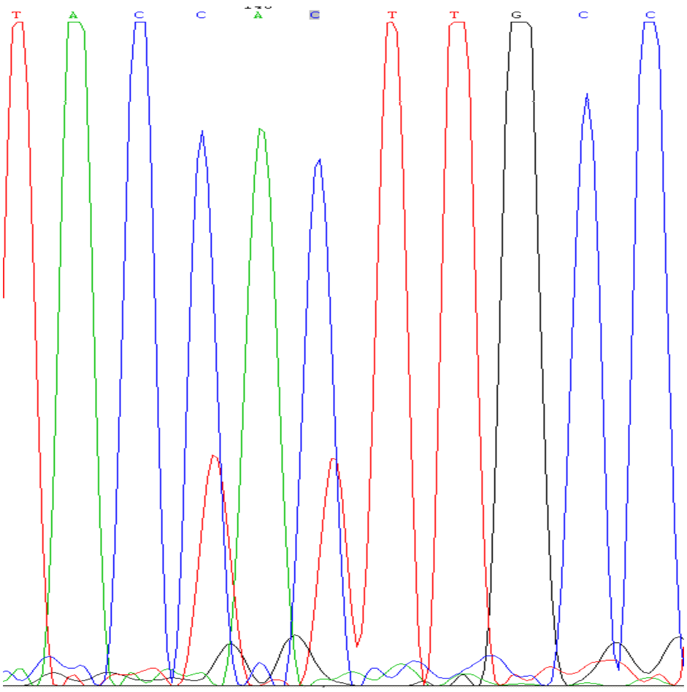

E18	1	CCCT	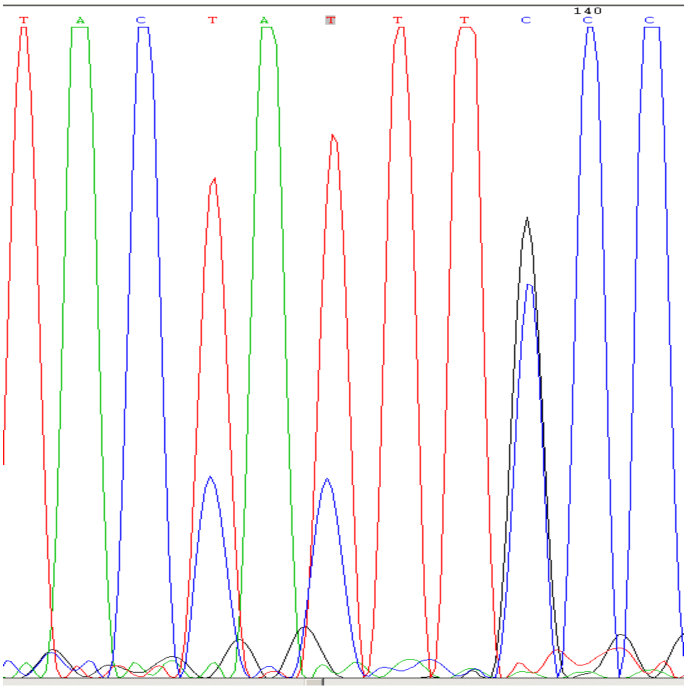
E19	1	CCCT	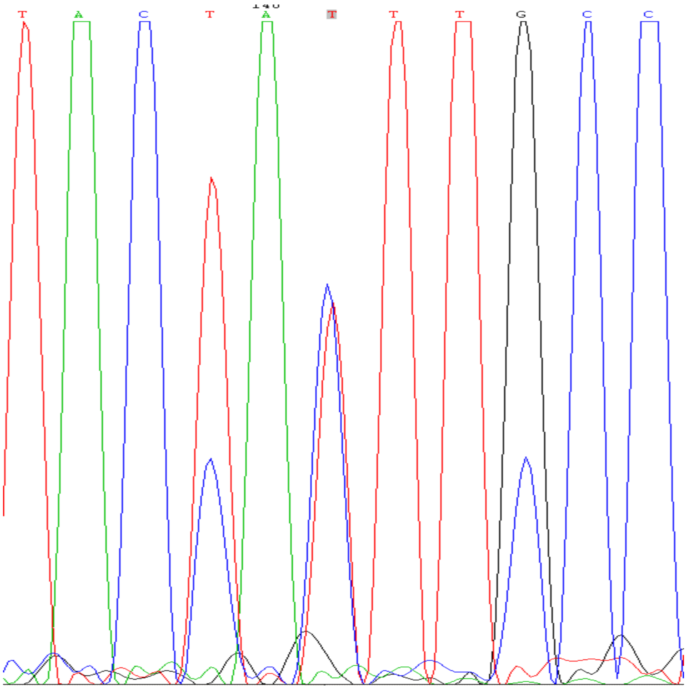

E20	1	CCCT	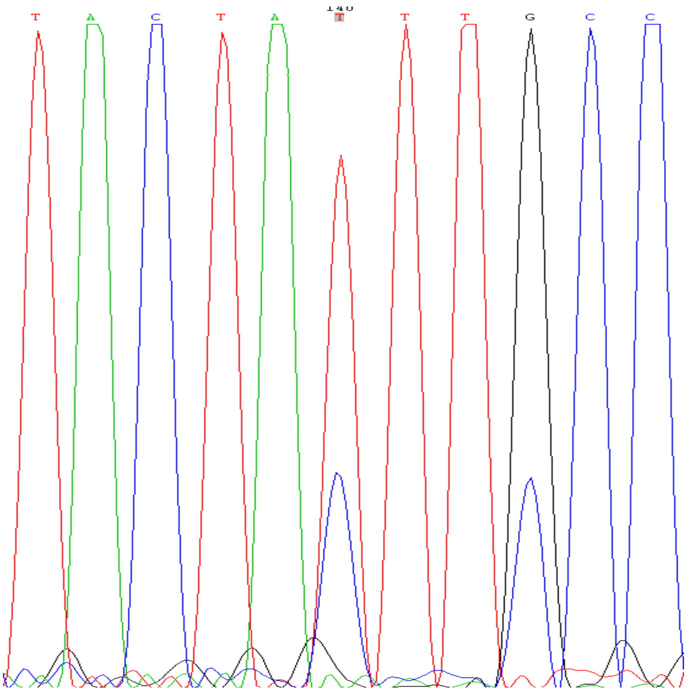
E21	0	CCCC	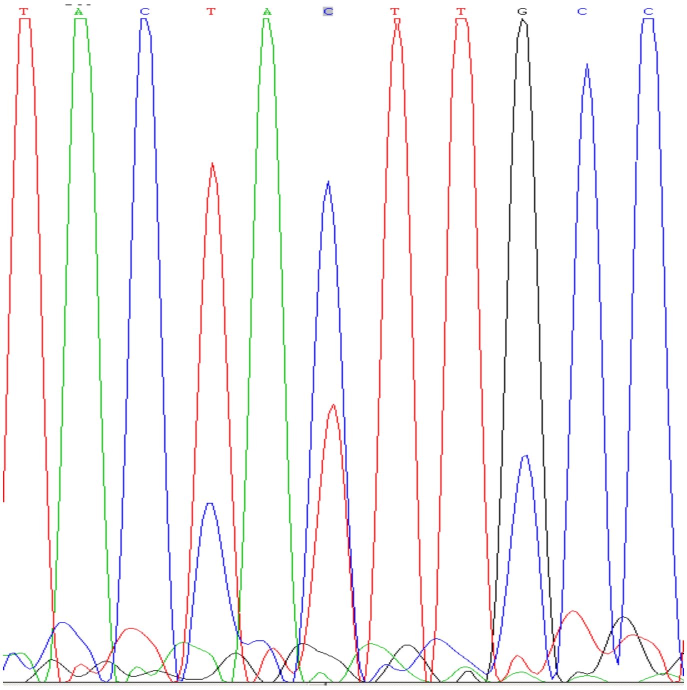

E22	1	CCCT	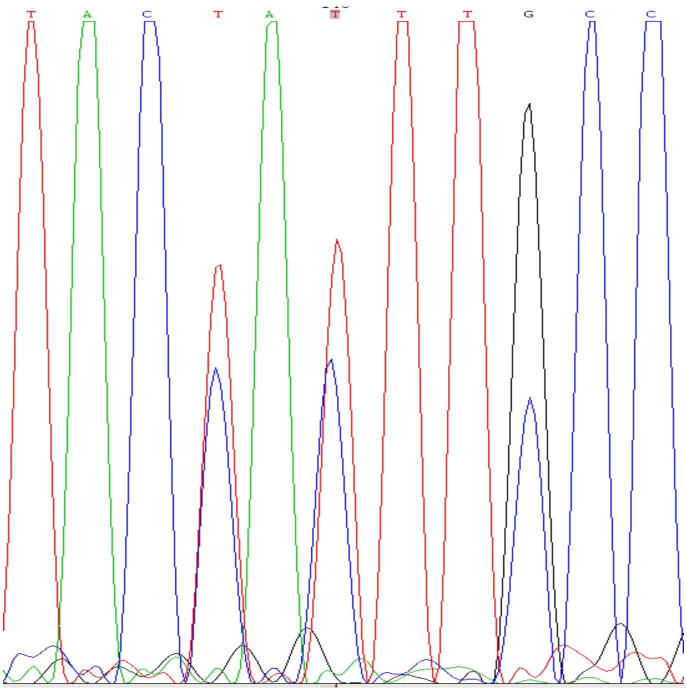
E23	1	CCCT	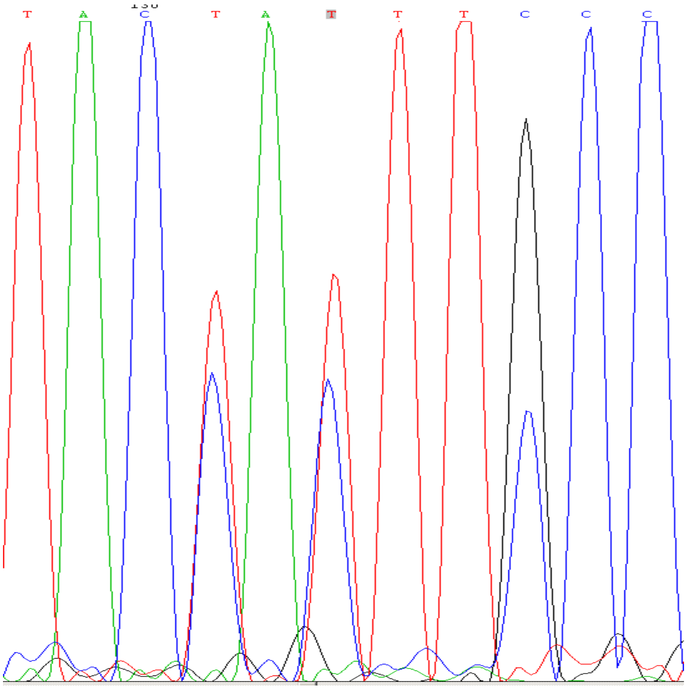

E24	1	CCCT	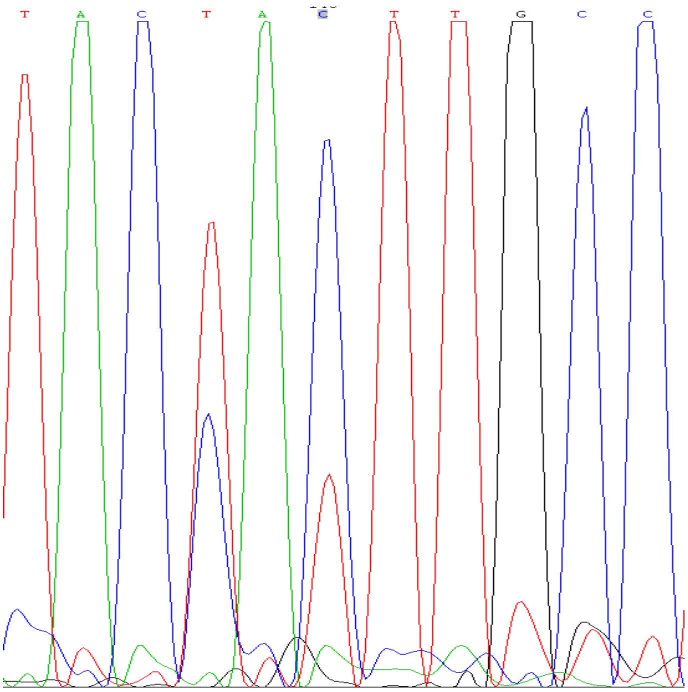
